# Supplementary material for: Validation study of bullous pemphigoid and pemphigus vulgaris recording in routinely collected electronic primary healthcare records in England
Source: BMJ Open. 2020 Jul 14;10(7):e035934. doi: 10.1136/bmjopen-2019-035934 (PMC7365488; doi:10.1136/bmjopen-2019-035934)
Supplement: Supplementary data [file bmjopen-2019-035934supp001.pdf]

Supporting information

Supplementary Table 1

The CPRD and HES algorithms used to determine the most likely blistering disease diagnosis were run using all entries for the blistering diseases described below. Based on clinical expertise, we determined Read and ICD-10 codes for bullous pemphigoid and pemphigus vulgaris. We also generated a list of "other blistering diseases" which could be confused with or be differential diagnoses for bullous pemphigoid or pemphigus vulgaris.

Supplementary Table 1. Blistering diseases and their associated Read or ICD-10 codes according to their disease category

Read code		ICD-10 code	
**Bullous pemphigoid**			
Specific codes			
M1450	Bullous pemphigoid	L12.0	Bullous pemphigoid
Broad codes			
M145.	Pemphigoid	L12.9	Pemphigoid, unspecified
M145z	Pemphigoid NOS		
**Pemphigus vulgaris**			
Specific codes			
M1445	Pemphigus vegetans	L10.1	Pemphigus vegetans
M1446	Pemphigus vulgaris	L10.0	Pemphigus vulgaris
Broad codes			
M144.	Pemphigus	L10.9	Pemphigus, unspecified
M144z	Pemphigus NOS		
**Other blistering diseases**			
2F6..11	O/E – pompholyx	L10.2	Pemphigus foliaceous
A900.17	Congenital syphilitic pemphigus	L10.3	Brazilian pemphigus
C371400	Porphyria cutanea tarda	L10.4	Pemphigus erythematosus
F4Cy100	Ocular pemphigoid	L10.5	Drug-induced pemphigus
M14..00	Bullous dermatoses	L10.8	Other pemphigus
M140.00	Dermatitis herpetiformis	L12.1	Cicatricial pemphigoid
M141.00	Subcorneal pustular dermatosis	L12.2	Chronic bullous disease of childhood

Continued

**Read code**		**ICD-10 code**	
M142.00	Juvenile dermatitis herpetiformis	L12.3	Acquired epidermolysis bullosa
M142.11	Juvenile pemphigoid	L12.8	Other pemphigoid
M144000	Benign pemphigus	L13.0	Dermatitis herpetiformis
M144200	Erythematous pemphigus	L13.1	Subcorneal pustular dermatitis
M144300	Foliaceous pemphigus	L13.8	Other specified bullous disorders
M144700	Wildfire pemphigus	L13.9	Bullous disorder, unspecified
M144800	Drug-induced pemphigus		
M145100	Benign pemphigus NOS		
M145200	Senile dermatitis herpetiformis		
M145300	Acquired epidermolysis bullosa		
M146.00	Benign mucous membrane pemphigoid		
M146000	Benign mucous membrane pemphigoid with no eye involvement		
M146011	Cicatricial pemphigoid		
M146100	Ocular pemphigoid		
M146z00	Benign mucous membrane pemphigoid NOS		
M14y.00	Other specified bullous dermatoses		
M14z.00	Bullous dermatoses NOS		
M151.00	Erythema multiforme		
M151.12	Toxic epidermal necrolysis		
M151700	Stevens-Johnson syndrome		
M151800	Toxic epidermal necrolysis		
M151y00	Other specified erythema multiforme		
M151z00	Erythema multiforme NOS		
M252100	Pompholyx unspecified		
M252200	Cheiropompholyx		
M252300	Podopompholyx		
M2y9000	Bullous cutaneous amyloidosis		
Myu1.00	[X]Bullous disorders		
Myu1000	[X]Other pemphigus		
Myu1200	[X]Other pemphigoid		
Myu1300	[X]Other specified bullous disorders		
Myu4100	[X]Other erythema multiforme		

Continued

Read code		ICD-10 code
PH33111	Benign familial chronic pemphigus	
PH3y200	Epidermolysis bullosa	
PH3y700	Epidermolysis bullosa simplex	
PH3y800	Epidermolysis bullosa letalis	
PH3y900	Epidermolysis bullosa dystrophica	
Pyu9100	[X]Other epidermolysis bullosa	
M2y9000	Bullous cutaneous amyloidosis	
Myu1.00	[X]Bullous disorders	
Myu1000	[X]Other pemphigus	
Myu1200	[X]Other pemphigoid	
Myu1300	[X]Other specified bullous disorders	
Myu4100	[X]Other erythema multiforme	
PH33111	Benign familial chronic pemphigus	
PH3y200	Epidermolysis bullosa	
PH3y700	Epidermolysis bullosa simplex	
PH3y800	Epidermolysis bullosa letalis	
PH3y900	Epidermolysis bullosa dystrophica	
Pyu9100	[X]Other epidermolysis bullosa	

Supplementary Figure 1

Flow diagram showing identification of incident cases of bullous pemphigoid and pemphigus vulgaris for inclusion in the validation study

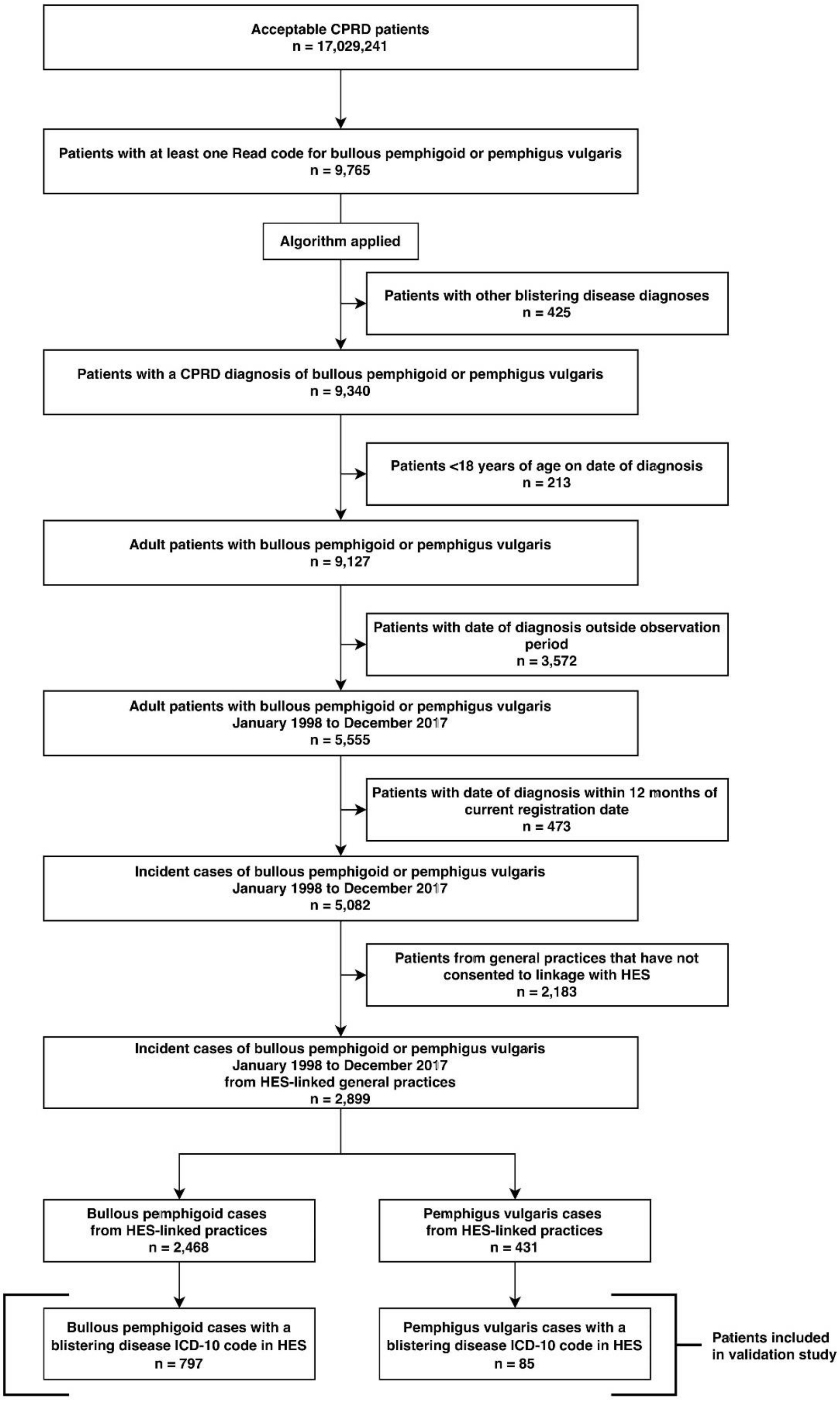

Supplementary Table 2

Supplementary Table 2. Blistering disease codes recorded as primary or secondary diagnoses in HES for the ten patients where the algorithm generated an uncertain diagnosis

**Blistering disease codes in HES**
**Specific + specific codes**
Bullous pemphigoid (L12.0) + pemphigus vulgaris (L10.0)
Bullous pemphigoid (L12.0) + cicatricial pemphigoid (L12.1)
**Specific + broad codes**
Bullous pemphigoid (L12.0) + pemphigus unspecified (L10.9)
Bullous pemphigoid (L12.0) + bullous disorder, unspecified (L13.9)
Dermatitis herpetiformis (L13.0) + bullous disorder, unspecified (L13.9)
Pemphigus vulgaris (L10.0) + other pemphigoid (L12.8)
Pemphigus vulgaris (L10.0) + pemphigus, unspecified (L10.9) + pemphigoid, unspecified (L12.9)
**Broad + broad codes**
Pemphigus, unspecified (L10.0) + pemphigoid, unspecified (L12.9)
