# Supplementary material for: Validation study of bullous pemphigoid and pemphigus vulgaris recording in routinely collected electronic primary healthcare records in England
Source: BMJ Open. 2020 Jul 14;10(7):e035934. doi: 10.1136/bmjopen-2019-035934 (PMC7365488; doi:10.1136/bmjopen-2019-035934)
Supplement: Author's manuscript [file bmjopen-2019-035934.draft_revisions.pdf]

BMJ Open

BMJ Open is committed to open peer review. As part of this commitment we make the peer review history of every article we publish publicly available.

When an article is published we post the peer reviewers' comments and the authors' responses online. We also post the versions of the paper that were used during peer review. These are the versions that the peer review comments apply to.

The versions of the paper that follow are the versions that were submitted during the peer review process. They are not the versions of record or the final published versions. They should not be cited or distributed as the published version of this manuscript.

BMJ Open is an open access journal and the full, final, typeset and author-corrected version of record of the manuscript is available on our site with no access controls, subscription charges or pay-per-view fees (http://bmjopen.bmj.com).

If you have any questions on BMJ Open's open peer review process please email info.bmjopen@bmj.com

BMJ Open

Validation of bullous pemphigoid and pemphigus vulgaris recording in routinely collected electronic primary healthcare records in England

Journal:	*BMJ Open*
Manuscript ID	bmjopen-2019-035934
Article Type:	Original research
Date Submitted by the Author:	22-Nov-2019
Complete List of Authors:	Persson, Monica; University of Nottingham Faculty of Medicine and Health Sciences, CEBD<br>Harman, Karen; University of Nottingham, Centre of Evidence Based Medicine<br>Vinogradova, Yana; University of Nottingham, Primary Care<br>Langan, Sinead; London School of Hygiene and Tropical Medicine, Department of Non-communicable Disease Epidemiology<br>Hippisley-Cox, Julia; University of Oxford, Nuffield Department of Primary Care Sciences<br>Thomas, Kim; University of Nottingham, Dermatology<br>Gran, Sonia; University of Nottingham, Centre of Evidence Based Dermatology
Keywords:	Dermatological epidemiology < DERMATOLOGY, DERMATOLOGY, EPIDEMIOLOGY

SCHOLARONE™
Manuscripts

I, the Submitting Author has the right to grant and does grant on behalf of all authors of the Work (as defined in the below author licence), an exclusive licence and/or a non-exclusive licence for contributions from authors who are: i) UK Crown employees; ii) where BMJ has agreed a CC-BY licence shall apply, and/or iii) in accordance with the terms applicable for US Federal Government officers or employees acting as part of their official duties; on a worldwide, perpetual, irrevocable, royalty-free basis to BMJ Publishing Group Ltd ("BMJ") its licensees and where the relevant Journal is co-owned by BMJ to the co-owners of the Journal, to publish the Work in this journal and any other BMJ products and to exploit all rights, as set out in our licence.

The Submitting Author accepts and understands that any supply made under these terms is made by BMJ to the Submitting Author unless you are acting as an employee on behalf of your employer or a postgraduate student of an affiliated institution which is paying any applicable article publishing charge ("APC") for Open Access articles. Where the Submitting Author wishes to make the Work available on an Open Access basis (and intends to pay the relevant APC), the terms of reuse of such Open Access shall be governed by a Creative Commons licence – details of these licences and which Creative Commons licence will apply to this Work are set out in our licence referred to above.

Other than as permitted in any relevant BMJ Author's Self Archiving Policies, I confirm this Work has not been accepted for publication elsewhere, is not being considered for publication elsewhere and does not duplicate material already published. I confirm all authors consent to publication of this Work and authorise the granting of this licence.

For peer review only

TITLE PAGE

Title

Validation of bullous pemphigoid and pemphigus vulgaris recording in routinely collected electronic primary healthcare records in England

Manuscript word, table, and figure counts

Word count: 2,947

Table count: 2

Figure count: 3

Names of authors, institutions

MSM Persson[1], KE Harman[1], Y Vinogradova[2], SM Langan[3], J Hippisley-Cox[4], KS Thomas[1], S Gran[1]

[1] Centre of Evidence Based Dermatology, University of Nottingham

[2] Division of Primary Care, University of Nottingham

[3] Department of Non-communicable Disease Epidemiology, London School of Hygiene and Tropical Medicine

[4] Nuffield Department of Primary Care Health Sciences, University of Oxford

Corresponding author

Monica SM Persson,

Centre of Evidence Based Dermatology, University Of Nottingham
King's Meadow Campus, Lenton Lane,
Nottingham
NG7 2NR
UK
monica.persson1@nottingham.ac.uk

Funding sources

This work was supported by the National Institute for Health Research (NIHR) Research for Patient Benefit grant (PB-PG-0817-20033). The views expressed are those of the authors and not necessarily those of the NIHR or the Department of Health and Social

For peer review only

Care. SML reports grants from Wellcome Senior Clinical Fellowship in Science (205039/Z/16/Z) during the conduct of the study.

Competing interests

MSM Persson, KE Harman, Y Vinogradova, SM Langan, J Hippisley-Cox, KS Thomas, S Gran declare no conflict of interest.

Keywords

Bullous pemphigoid, Pemphigus vulgaris, Validation, Read code, Electronic healthcare records

Data availability

Data can be requested from www.cprd.com

Patient and public involvement

The study had patient and public involvement in the design and interpretation of the work.

For peer review only

ABSTRACT

Objectives

The validity of bullous pemphigoid and pemphigus vulgaris recording in routinely collected healthcare data in the UK is unknown. We assessed the positive predictive value (PPV) for bullous pemphigoid and pemphigus vulgaris primary care Read codes in the Clinical Practice Research Datalink (CPRD) using linked inpatient data (Hospital Episode Statistics; HES) as the diagnostic benchmark.

Setting

Adult participants with bullous pemphigoid or pemphigus vulgaris registered with HES-linked general practices in England between January 1998 to December 2017. Code-based algorithms were used to identify patients from the CPRD and extract their benchmark blistering disease diagnosis from HES.

Primary outcome measure

The PPVs of Read codes for bullous pemphigoid and pemphigus vulgaris.

Results

Of 2,468 incident cases of bullous pemphigoid and 431 of pemphigus vulgaris, 797 and 85 patients, respectively, had a hospitalisation record for a blistering disease. The PPV for bullous pemphigoid Read codes was 93.2% (95%CI 91.3 to 94.8). Of the bullous pemphigoid cases, 3.0% had a HES diagnosis of pemphigus vulgaris and 3.8% of another blistering disease. The PPV for pemphigus vulgaris Read codes was 58.5% (95%CI 48.0 to 68.9%). Of the pemphigus vulgaris cases, 24.7% had a HES diagnosis of bullous pemphigoid and 16.5% of another blistering disease.

Conclusions

The CPRD can be used to study bullous pemphigoid, but recording of pemphigus vulgaris needs to improve in primary care.

ARTICLE SUMMARY - STRENGTHS AND LIMITATIONS OF THIS STUDY

- This is the first study to validate the recording of bullous pemphigoid and pemphigus vulgaris in primary healthcare records in England

For peer review only

- The study involved a large sample size and the application of algorithms to identify both primary care and benchmark hospital inpatient diagnoses
- Blistering diagnoses recorded in hospital inpatient records were regarded as the benchmark diagnosis, but may be subject to misclassification

MAIN TEXT

INTRODUCTION

Bullous pemphigoid and pemphigus vulgaris are rare autoimmune blistering diseases characterised by blistering of the skin and mucous membrane and associated with high mortality.[1,2] There is a continued need to understand the causes, natural history, and disease associations in order to better inform patients and plan treatment. However, there is a scarcity of recent epidemiological research on these distinct diseases in the UK and many unanswered questions. Furthermore, interpretation of available evidence is limited by the poor external validity and lack of power that are a feature of hospital-based studies.[3-6]

Routinely collected electronic healthcare records (EHRs) may offer an excellent opportunity to examine bullous pemphigoid and pemphigus vulgaris at population-level due to the availability of data on a large number of patients that are broadly representative of the UK population.[7] Furthermore, the presence of prescription data allows the important association between possible drug exposure and bullous pemphigoid to be established, and longitudinal follow-up allows long-term outcomes to be assessed.[8] However, as EHRs were created for clinical purposes, and not directly for research, it is essential to establish the validity of the diagnoses of interest.[9]

Diagnoses of bullous pemphigoid or pemphigus vulgaris are usually made in secondary care and entered into the primary care records by general practitioners (GPs) or other practice staff using Read codes. Read codes have been used previously to identify patients with bullous pemphigoid and pemphigus vulgaris in EHRs, but have not been validated [2,8,10,11]. A previous study examined the incidence of bullous pemphigoid and pemphigus vulgaris in the UK and found that the average age of patients with

For peer review only

pemphigus vulgaris was older than observed in other studies.[2] Furthermore it coincided with the peak age for bullous pemphigoid, suggesting that pemphigus vulgaris Read codes may not be accurate and that, in some cases, bullous pemphigoid was misclassified as pemphigus vulgaris.

Due to the linkage between primary and secondary care data, we now have the opportunity to externally validate bullous pemphigoid and pemphigus vulgaris Read codes. This study aimed to determine the positive predictive value (PPV) for bullous pemphigoid and pemphigus vulgaris primary care diagnostic Read codes in Clinical Practice Research Datalink (CPRD) using linked inpatient data (Hospital Episode Statistics; HES) as the benchmark.

For peer review only

METHODS

Study design

This was a cohort study to validate Read codes for bullous pemphigoid and pemphigoid vulgaris in the CPRD. We followed guidelines for the reporting of studies conducted using observational routinely collected health data.[12]

Data sources

CPRD

The CPRD GOLD is a longitudinal database of UK general practices using the Vision software system. It contains anonymised healthcare records from 1987 onwards for approximately 17 million patients with a current coverage of approximately 2.7 million (4%) of the UK population. Routinely collected data within the CPRD include demographic and clinical information.[7] Symptoms and diagnoses, generated from primary care consultations or obtained from hospital discharge or specialist clinic letters, are entered by GPs or other practice staff using Read codes.[13]

The data in the CPRD have repeatedly been shown to be of good research quality.[14] At a practice-level, participating practices are assigned an “up-to-standard” date on completion of regular audits confirming data quality. At the patient-level, records are

assessed and patients are deemed “acceptable” if data checks indicate that their record meets pre-specified quality standards.

Hospital Episode Statistics (HES)

HES admitted patient care data are available for approximately 75% of English practices, covering over 10 million patients, that have consented to provide patient-level information from linked resources.[15] HES admitted patient care contains demographic and clinical data, coded using the International Classification of Diseases version 10 (ICD-10)[16,17] Each hospital episode is attributed one primary diagnosis, whilst comorbidities and other diagnoses are recorded as secondary diagnoses.

Study population

Adult men and women registered with 410 HES-linked general practices in England during the period of 1 January 1998 to 31 December 2017 were included.

Case definition of bullous pemphigoid and pemphigus vulgaris

Diagnostic Read code lists for bullous pemphigoid and pemphigus vulgaris were developed based on the expert opinion of two consultant dermatologists (KEH and SML) and a GP (JHC). Bullous pemphigoid Read codes were classed as specific (M145000, “bullous pemphigoid”) or broad (M145.00, “pemphigoid”; M145z00, “pemphigoid not otherwise specified”). Likewise, pemphigus vulgaris codes were grouped as specific (M144600, “pemphigus vulgaris”; M144500, “pemphigus vegetans”) or broad (M144.00, “pemphigus”; M144z00, “pemphigus not otherwise specified”).

Clinical data were sought for all patients that had at least one specific or broad Read code for bullous pemphigoid or pemphigus vulgaris. Based on clinical experience, it was considered inappropriate to define a case based on the first recording of bullous pemphigoid or pemphigus vulgaris without taking into account subsequent Read code entries for blistering diseases which were sometimes multiple and conflicting. A code-based algorithm (Figure 1) was therefore applied to determine the most likely diagnosis. The date of diagnosis was taken as the first recording of the Read code generated through the algorithm.

For peer review only

Observation period

The observation period commenced on the latest of (i) 1st January 1998, (ii) the patient's 18th birthday, (iii) 1 year after the patient was registered with their current general practice, or (iv) the practice's up-to-standard date. A 1-year lag period from the patient's registration date with their GP was imposed to minimise the risk of prevalent cases being identified as incident cases.[18]

The observation period terminated on the earliest of (i) 31st December 2017, (ii) the date of death, (iii) the date the patient left the practice, (iv) the practice's last data collection date, (v) the most recent linkage date between CPRD and HES.

Validation of bullous pemphigoid and pemphigus vulgaris Read codes

For people with a bullous pemphigoid or pemphigus vulgaris diagnosis in primary care, we identified those with a hospital inpatient episode recording a blistering disease (ICD-10: L10, L12, L13) as a primary or secondary diagnosis at any point. Due to the presence of multiple records per patient, an algorithm based on clinical knowledge (Figure 2) was applied to determine one secondary care ICD-10 blistering disease diagnosis per patient, which was regarded as their benchmark diagnosis. The date of diagnosis was taken as the first recording of the diagnosis generated through the algorithm. The distribution of the benchmark diagnoses was examined for bullous pemphigoid and pemphigus vulgaris cases identified in the CPRD.

ICD-10 codes L12.0 (bullous pemphigoid) and L12.9 (pemphigoid, unspecified) were classed as "bullous pemphigoid". ICD-10 codes L10.0 (pemphigus vulgaris), L10.1 (pemphigus vegetans), and L10.9 (pemphigus, unspecified) were classed as "pemphigus vulgaris". All other blistering disease ICD-10 codes and where the HES algorithm generated an uncertain diagnosis were classed as "other". See Supplementary Table 1 for the full list of blistering disease codes.

Statistical analysis

The sex and age of bullous pemphigoid and pemphigus vulgaris cases were presented descriptively. Age was assessed for normality visually, the mean (standard deviation,

For peer review only

SD) or median (interquartile range, IQR) were calculated as appropriate. For bullous pemphigoid and pemphigus vulgaris cases with a hospital inpatient record for a blistering disease, the PPV and associated 95% confidence interval (CI) were calculated for the specific, broad, and combined Read codes for bullous pemphigoid and pemphigus vulgaris against their benchmark diagnosis from HES. The blistering disease secondary care diagnoses were presented descriptively. Analyses were conducted using Stata 16 (StataCorp LP).

Sensitivity analysis

The most recent Read code was used for patients with conflicting Read codes (i.e., patients who did not have a more commonly occurring Read code; last step of algorithm, Figure 1). This was done under the assumption that diagnoses are likely to be refined over time and the most recent code was likely to be most accurate. The impact of this assumption was investigated through a sensitivity analysis comparing the PPVs for bullous pemphigoid and pemphigus vulgaris in all cases versus excluding patients with conflicting Read codes.

Ethical approval

The present study was approved by the Independent Scientific Advisory Committee for the CPRD (ISAC protocol no 18_224).

Results

Study population

A total of 2,899 incident cases of bullous pemphigoid and pemphigus vulgaris were identified in HES-linked practices using the algorithm. The median (IQR) age at diagnosis was 81 years (73 to 87) for the 2,468 bullous pemphigoid cases, and 64 years (48 to 77) for the 431 pemphigus vulgaris cases. The peak age for bullous pemphigoid recording was around 80-84 years. In contrast, two peaks were observed for pemphigus vulgaris case recording, one at 50-54 years and a later, slightly higher, peak at 75-79 years (Figure 3).

For peer review only

The proportion of women were 56.7% and 64.5% for bullous pemphigoid and pemphigus vulgaris, respectively. Of the 2,899 cases, 223 (9.0%) had at least one conflicting Read code indicative of another blistering disease or a general blistering disease code (e.g., "bullous dermatoses").

Validation of bullous pemphigoid and pemphigus vulgaris diagnostic codes

Of 2,899 incident cases of bullous pemphigoid or pemphigus vulgaris, 882 (30.4%) had at least one hospital episode recording a blistering disease diagnosis. In 44.0% of these patients, it was a primary diagnosis. Using the algorithm, ten patients (1.1%) had an uncertain diagnosis and were classified as "other". The blistering disease codes of these cases are shown in Supplementary Table 2.

The distribution of HES diagnoses for the bullous pemphigoid and pemphigus vulgaris incident cases and PPV for bullous pemphigoid and pemphigus vulgaris Read codes are shown in Table 1.

Table 1. Distribution of benchmark blistering disease diagnoses for bullous pemphigoid and pemphigus vulgaris incident cases identified in the CPRD, shown with the PPV (95%CI) for specific, broad, and combined Read codes for each disease

CPRD Read code	Hospital episode diagnosis (benchmark)			n	% PPV (95%CI)
	Bullous pemphigoid	Pemphigus vulgaris	Other		
**Bullous pemphigoid**					
Specific (M145000)	527 (95.6%)	12 (2.2%)	12 (2.2%)	551	95.6 (93.6 to 97.1)
Broad (M145.00, M145z00)	216 (87.8%)	12 (4.9%)	18 (7.3%)	246	87.8 (83.1 to 91.4)
Total	744 (93.2%)	24 (3.0%)	30 (3.8%)	797	93.2 (91.3 to 94.8)
**Pemphigus vulgaris**					
Specific (M144600, M144500)	2 (9.1%)	18 (81.8%)	2 (9.1%)	22	81.8 (58.8 to 93.4)
Broad (M144.00, M144z00)	19 (30.2%)	32 (50.8%)	12 (19.1%)	63	50.8 (38.4 to 63.1)
Total	21 (24.7%)	50 (58.8%)	14 (16.5%)	85	58.8 (48.0 to 68.9)

ICD-10 codes L10.0 (pemphigus vulgaris), L10.1 (pemphigus vegetans), and L10.9 (pemphigus, unspecified) are classed as a hospital episode diagnosis of "pemphigus vulgaris". ICD-10 codes L12.0 (bullous pemphigoid) and L12.9 (pemphigoid, unspecified) are classed as "bullous pemphigoid". All other blistering disease ICD-10 codes and where the HES algorithm generated an uncertain diagnosis (n=7 for bullous pemphigoid and n=3 for pemphigus vulgaris) are classed as "other"

For peer review only

Bullous pemphigoid

Of 2,468 incident cases, 797 (32.2%) had a hospitalisation record for a blistering disease. Cases were most commonly coded with specific (69.1%) rather than broad (30.8%) Read codes. The PPV of the bullous pemphigoid Read codes combined was 93.2% (95%CI 91.3 to 94.8). Broad Read codes had a lower PPV (87.8%, 95%CI 83.1 to 91.4) than the specific codes (95.6%, 95%CI 93.6 to 97.1).

Pemphigus vulgaris

Of 431 incident cases, 85 (20%) had a hospitalisation record for a blistering disease. Cases were more commonly coded with broad disease codes (74.1%) than specific codes (25.9%). The PPV of the pemphigus vulgaris Read codes combined was 58.8% (95%CI 48.0 to 68.9). Specific Read codes showed a higher PPV (81.8%, 95%CI 58.8 to 93.4) than broad codes (50.8%, 95%CI 38.4 to 63.1).

Sensitivity analysis

The sensitivity analyses excluding the 223 patients with conflicting Read codes resulted in broadly unchanged PPVs. The PPV for the 776 patients with consistent bullous pemphigoid Read codes was 93.6% (95%CI 91.6 to 95.1). The PPV for the 75 patients with consistent pemphigus vulgaris Read codes was 61.3% (95%CI 49.7 to 71.8).

Inconsistent codes

Bullous pemphigoid

A benchmark, secondary care diagnosis of pemphigus vulgaris or another blistering disease diagnosis was made for 24 (3.0%) and 30 (3.8%) of the 797 cases, respectively. The most common "other" benchmark diagnosis was cicatricial pemphigoid (n=11, 36.7%; L12.1, mucous membrane pemphigoid). Other benchmark diagnoses were other pemphigus (L10.8), other pemphigoid (L12.8), and bullous disorder unspecified (L13.9).

Pemphigus vulgaris

Of the 85 cases with a primary care diagnosis for pemphigus vulgaris, 21 (24.7%) had a HES diagnosis for bullous pemphigoid and 14 (16.5%) for another blistering diagnosis. The most common "other" secondary care diagnosis was for pemphigus foliaceous (n=6,

For peer review only

42.9%; L10.2). Other benchmark diagnosis were drug-induced pemphigus (L10.5), cicatricial pemphigoid (L12.1), dermatitis herpetiformis (L13.0), subcorneal pustular dermatitis (L13.1), and other specified bullous disorder (L13.8).

DISCUSSION

Main findings

Incident diagnoses of bullous pemphigoid and pemphigus vulgaris identified in the CPRD were compared to the blistering disease diagnoses made in secondary care (benchmark diagnosis). Overall, 882 (30.4%) patients identified in the CPRD also had hospitalisation records for a blistering disease. We found good consistency between primary and secondary care diagnoses for bullous pemphigoid and a high PPV for bullous pemphigoid Read codes (93.2%). The PPV for the specific Read codes for bullous pemphigoid was higher (95.6%) than that of the broad Read codes (87.8%) and captured a greater proportion of cases (69.1%). The consistency was not as good for pemphigus vulgaris, with a moderate PPV for pemphigus vulgaris Read codes (58.8%). The majority of cases of pemphigus vulgaris were coded with broad disease codes (74.1%) with lower PPVs (50.8%). The PPV for the specific pemphigus vulgaris Read codes was higher (81.8%), but captured only a subset of the population (25.9%).

Our study shows that the codes for bullous pemphigoid in the CPRD appear to be capturing people who have the disease and cases reflect expected age distributions. Approximately 93% of bullous pemphigoid cases identified using the algorithm are likely to have bullous pemphigoid. In contrast, only 59% of pemphigus vulgaris cases identified are likely to have pemphigus vulgaris, with almost 25% probably suffering from bullous pemphigoid. The probable misclassification of bullous pemphigoid cases as pemphigus vulgaris likely explains the peak of pemphigus vulgaris codes observed at 75-79 years, coinciding with the peak age for bullous pemphigoid. Consequently, the use of data from the CPRD for pemphigus vulgaris research may be limited.

For peer review only

Comparability with other studies

To date, no studies have validated Read codes and only two studies have validated ICD codes for bullous pemphigoid or pemphigus vulgaris using EHRs (Table 2). The PPV for bullous pemphigoid Read codes in the CPRD is comparable to that found in routinely collected healthcare data from Sweden (National Patient Register; 92%), which validated bullous pemphigoid ICD-10 codes using information from pathology registers and medical records.[19] In contrast, the PPVs for bullous pemphigoid and pemphigus vulgaris Read codes were lower in the CPRD than those presented for the electronic medical records in the US (Northwestern Medicine Electronic Data Warehouse; 99% for bullous pemphigoid and 100% for pemphigus ICD-9 codes)[20]. However, Hsu et al[20] presented the PPV only for those cases with multiple disease codes for the same disease, and not for those with a single code or those with conflicting codes. The stringency of the case definition may explain the higher PPV found, but would not be suitable if the study aim was to identify all (or nearly all) cases in a population-based study. Furthermore, the work included several subsets of pemphigus disease, rather than just pemphigus vulgaris, and did not provide any indication of potential misclassification between different pemphigus diseases. Further comparisons between our study and the aforementioned studies are hindered as the studies validated ICD-9 and ICD-10 codes rather than Read codes. However, the evidence indicates that routinely collected healthcare data may be a valuable source for investigating bullous pemphigoid. The role of such data sources for investigating pemphigus vulgaris remains uncertain.

For peer review only

Table 2. Published validation studies of bullous pemphigoid or pemphigus vulgaris diagnostic codes in HER

Publication	Disease	Classification	EHR	Population	Validation method	PPV (95%CI)
**Hsu 2016**	Pemphigus* (694.4) and bullous pemphigoid (694.5)	ICD-9 Clinical Modification diagnostic codes	Northwestern Medicine Electronic Data Warehouse; Inpatient + outpatient data	Patients with multiple codes for either pemphigus (n=161) or bullous pemphigoid (n=126).	Manual review of outpatient and inpatient records. Diagnosis was confirmed by ≥2 indicators of disease:<br>• Clinical diagnosis<br>• Biopsy histology<br>• Direct immunofluorescence<br>• Indirect immunoflourescence or enzyme-linked immunosorbent assay.	Pemphigus: 100% (96 to 100);<br>Bullous pemphigoid: 99% (93 to 99)
**Grönhagen 2017**	Bullous pemphigoid (L12.0, L12.8, L12.9)	ICD-10 diagnostic codes	Swedish National Patient Register; Inpatient + outpatient data	307 patients with primary or secondary diagnosis of bullous pemphigoid who had medical records available for review	Manual review of immunopathological and histopathological registries and medical records. Diagnosis was confirmed by:<br>• Clear clinical history and physical examination, and<br>• Positive enzyme-linked immunosorbent assay, or<br>• Histopathological or immunopathological pattern consistent with bullous pemphigoid	92%

* Pemphigus vulgaris, pemphigus vegetans, pemphigus foliaceus, pemphigus erythematosus, paraneoplastic pemphigus, and drug induced pemphigus

Strengths and limitations

The strengths of the present work include a large sample size and the application of algorithms to identify both primary care and benchmark hospital inpatient diagnoses. One-off occurrences of a bullous pemphigoid or pemphigus vulgaris code in the presence of alternate blistering disease codes may indicate a working diagnosis that is later revised or may be incorrect due to a typographical error or oversight. Based on clinical knowledge, using an algorithm that prioritised diagnoses with multiple entries and consistent coding was considered to help reduce the impact of recording errors.

The study is limited by the inability to calculate the negative predictive value, sensitivity, and specificity of the Read codes. Additionally, HES diagnoses were regarded as the

For peer review only

benchmark in the present study, but may be inaccurate. Nevertheless, this was regarded as the best approach as external validation with hospital notes was not possible. Finally, validation was only possible for a third of patients and it may be that patients with a hospitalisation record for a blistering disease are not representative of the overall population of patients as they are more likely to have severe disease and associated comorbidities.

Possible explanations for findings

Almost a quarter of patients with Read codes indicative of pemphigus vulgaris in the CPRD may have bullous pemphigoid. Such misclassification may be due to unfamiliarity with the disease by GPs and administrators or typographical error. Pemphigus and pemphigoid sound similar and, to those unfamiliar with the diseases, the terms may be thought to be interchangeable. Additionally, the lower prevalence of pemphigus vulgaris in the population might explain why lower PPVs were reported for pemphigus vulgaris than bullous pemphigoid.

Inclusion of broad Read codes also contribute to the apparent misclassification seen. For example, although we have assumed that broad pemphigoid codes identify a bullous pemphigoid patient such a code could correctly be attributed to a patient with a different pemphigoid disease, such as mucous membrane pemphigoid. Similarly, a patient with pemphigus foliaceous could correctly be attributed a broad pemphigus code. Correct attribution of broad Read codes to patients with pemphigoid or pemphigus diseases other than bullous pemphigoid or pemphigus vulgaris likely explain the lower PPVs seen for the broad versus specific Read codes.

Possible implications for future research

Implementation of an algorithm allows a consistent and reproducible approach to identifying patients in the presence of multiple, sometimes conflicting, blistering disease codes. The algorithm was developed based on clinical expertise and future research should be conducted to test alternate approaches and determine the sensitivity, specificity, and negative predictive value.

For peer review only

The discrepant codes observed complicates the examination of pemphigus vulgaris in the CPRD, and may also underestimate the true incidence of bullous pemphigoid. However, only a minor proportion of the population with a benchmark diagnosis for bullous pemphigoid (<3%) were miscoded as pemphigus vulgaris in primary care, and the effect may therefore be minimal.

Conclusion

The PPV of Read codes for bullous pemphigoid are high in the CPRD, but only moderate for pemphigus vulgaris. The CPRD is a useful data source for bullous pemphigoid research, but may have lower utility for pemphigus vulgaris. We suggest future studies of bullous pemphigoid in the CPRD should consider using this study's validated Read code list to identify cases. EHRs, such as the CPRD, could be an invaluable resource for the examination of a rare disease such as pemphigus vulgaris, but there is a need for more accurate recording of diagnoses in primary care.

ACKNOWLEDGMENTS

None

AUTHOR CONTRIBUTIONS

MSMP, KEH, YV, SML, JHC, KST, and SG contributed to the conception and design. MSMP, YV, and SG contributed to data acquisition and analysis. MSMP, KEH, YV, SML, JHC, KST, and SG contributed to the interpretation of the data. MSMP drafted the manuscript and KEH, YV, SML, JHC, KST, and SG critically revised the manuscript. SG is the guarantor of the work.

REFERENCES

1 Kridin K, Schwartz N, Cohen AD *et al.* Mortality in bullous pemphigoid: A systematic review and meta-analysis of standardized mortality ratios. *The Journal of Dermatology* 2018; **45**: 1094-100.
2 Langan S, Smeeth L, Hubbard R *et al.* Bullous pemphigoid and pemphigus vulgaris—incidence and mortality in the UK: population based cohort study. *Bmj* 2008; **337**: a180.
3 Gudi VS, White MI, Cruickshank N *et al.* Annual incidence and mortality of bullous pemphigoid in the Grampian Region of North-east Scotland. *Br J Dermatol* 2005; **153**: 424-7.
4 Lloyd-Lavery A, Chi C-C, Wojnarowska F *et al.* The Associations Between Bullous Pemphigoid and Drug Use: A UK Case-Control Study. *JAMA Dermatology* 2013; **149**: 58-62.

For peer review only

5 Taghipour K, Chi C-C, Vincent A *et al.* The Association of Bullous Pemphigoid With Cerebrovascular Disease and Dementia: A Case-Control Study. *JAMA Dermatology* 2010; **146**: 1251-4.
6 Saha M, Bhogal B, Black MM *et al.* Prognostic factors in pemphigus vulgaris and pemphigus foliaceus. *Br J Dermatol* 2014; **170**: 116-22.
7 Herrett E, Gallagher AM, Bhaskaran K *et al.* Data resource profile: clinical practice research datalink (CPRD). *International journal of epidemiology* 2015; **44**: 827-36.
8 Douros A, Rouette J, Yin H *et al.* Dipeptidyl Peptidase-4 Inhibitors and the Risk of Bullous Pemphigoid Among Patients With Type 2 Diabetes. *Diabetes care* 2019: dc190409.
9 Manuel DG, Rosella LC, Stukel TA. Importance of accurately identifying disease in studies using electronic health records. *BMJ* 2010; **341**: c4226.
10 Langan SM, Hubbard R, Fleming K *et al.* A population-based study of acute medical conditions associated with bullous pemphigoid. *British Journal of Dermatology* 2009; **161**: 1149-52.
11 Langan SM, Groves RW, West J. The relationship between neurological disease and bullous pemphigoid: a population-based case–control study. *Journal of Investigative Dermatology* 2011; **131**: 631-6.
12 Benchimol EI, Manuel DG, To T *et al.* Development and use of reporting guidelines for assessing the quality of validation studies of health administrative data. *Journal of Clinical Epidemiology* 2011; **64**: 821-9.
13 Chisholm J. The Read clinical classification. *BMJ: British Medical Journal* 1990; **300**: 1092.
14 Herrett E, Thomas SL, Schoonen WM *et al.* Validation and validity of diagnoses in the General Practice Research Database: a systematic review. *British journal of clinical pharmacology* 2010; **69**: 4-14.
15 CPRD. Release Notes: CPRD GOLD June 2019. In: (Agency MHpR, ed). 2019.
16 WHO. International statistical classification of diseases and related health problems: 10th revision (ICD-10). *http://www. who. int/classifications/apps/icd/icd* 1992.
17 Herbert A, Wijlaars L, Zylbersztejn A *et al.* Data Resource Profile: Hospital Episode Statistics Admitted Patient Care (HES APC). *International Journal of Epidemiology* 2017; **46**: 1093-i.
18 Lewis JD, Bilker WB, Weinstein RB *et al.* The relationship between time since registration and measured incidence rates in the General Practice Research Database. *Pharmacoepidemiology and drug safety* 2005; **14**: 443-51.
19 Grönhagen C, Nilzen K, Seifert O *et al.* Bullous Pemphigoid: Validation of the National Patient Register in two counties in Sweden, 2001 to 2012. *Acta dermato-venereologica* 2017; **97**: 32-5.
20 Hsu D, Brieva J, Nardone B *et al.* Validation of database search strategies for the epidemiological study of pemphigus and pemphigoid. *The British journal of dermatology* 2016; **174**: 645.

For peer review only

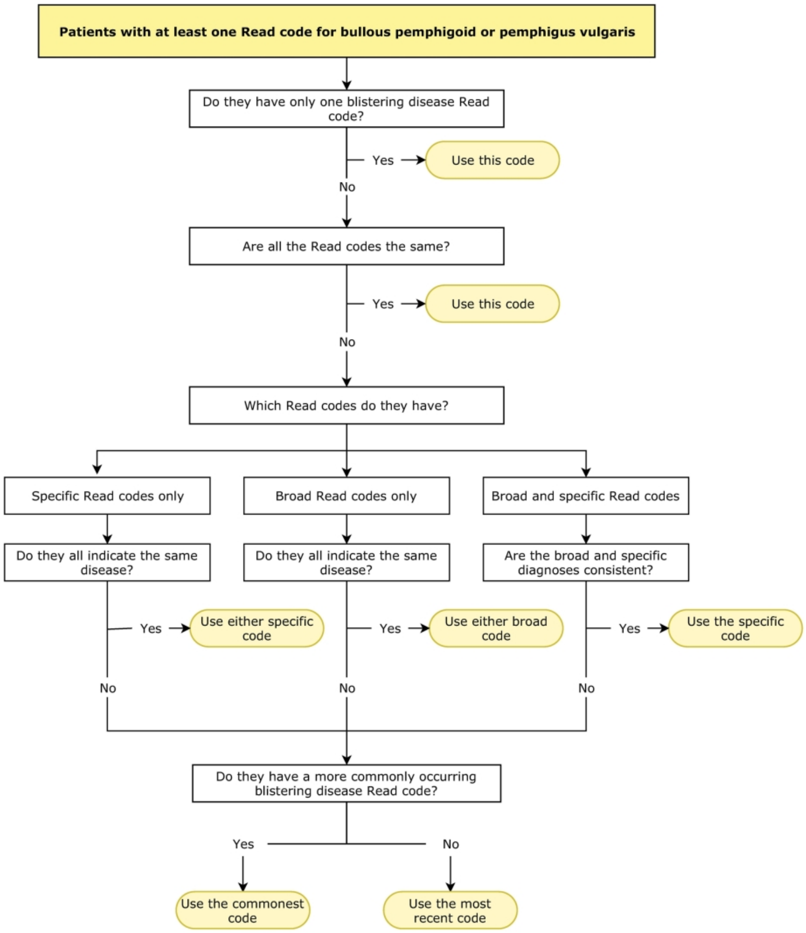

Figure 1. The code-based algorithm used to determine a diagnosis of bullous pemphigoid or pemphigus vulgaris using Read codes in the CPRD. The date of diagnosis was taken as the first recording of the Read code generated through the algorithm

210x296mm (300 x 300 DPI)

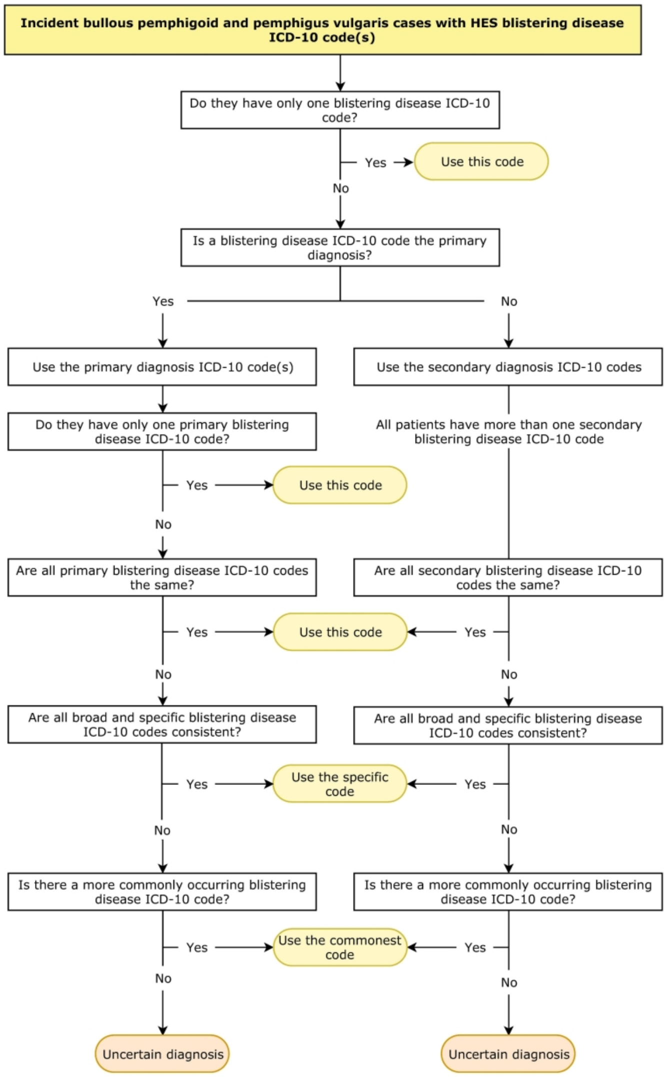

Figure 2. Code-based algorithm used to determine a benchmark diagnosis from secondary care HES data in patients identified from CPRD as having bullous pemphigoid or pemphigus vulgaris. The date of diagnosis was taken as the first recording of the diagnosis generated through the algorithm

210x296mm (300 x 300 DPI)

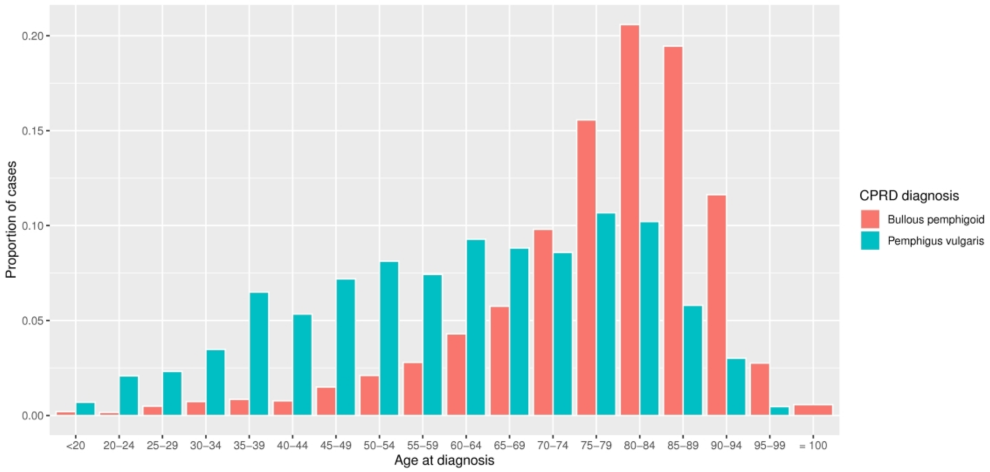

Figure 3. Age at diagnosis (years) in patients with bullous pemphigoid (n=2,468) and pemphigus vulgaris (n=431)

285x135mm (300 x 300 DPI)

Supporting information

Supplementary Table 1

Supplementary Table 1. Blistering diseases and their associated Read or ICD-10 codes according to their disease category

Read code		ICD-10 code	
**Bullous pemphigoid**			
Specific codes			
M1450	Bullous pemphigoid	L12.0	Bullous pemphigoid
Broad codes			
M145.	Pemphigoid	L12.9	Pemphigoid, unspecified
M145z	Pemphigoid NOS		
**Pemphigus vulgaris**			
Specific codes			
M1445	Pemphigus vegetans	L10.1	Pemphigus vegetans
M1446	Pemphigus vulgaris	L10.0	Pemphigus vulgaris
Broad codes			
M144.	Pemphigus	L10.9	Pemphigus, unspecified
M144z	Pemphigus NOS		
**Other blistering diseases**			
2F6..11	O/E – pompholyx	L10.2	Pemphigus foliaceous
A900.17	Congenital syphilitic pemphigus	L10.3	Brazilian pemphigus
C371400	Porphyria cutanea tarda	L10.4	Pemphigus erythematosus
F4Cy100	Ocular pemphigoid	L10.5	Drug-induced pemphigus
M14..00	Bullous dermatoses	L10.8	Other pemphigus
M140.00	Dermatitis herpetiformis	L12.1	Cicatricial pemphigoid
M141.00	Subcorneal pustular dermatosis	L12.2	Chronic bullous disease of childhood
M142.00	Juvenile dermatitis herpetiformis	L12.3	Acquired epidermolysis bullosa
M142.11	Juvenile pemphigoid	L12.8	Other pemphigoid
M144000	Benign pemphigus	L13.0	Dermatitis herpetiformis
M144200	Erythematous pemphigus	L13.1	Subcorneal pustular dermatitis
M144300	Foliaceous pemphigus	L13.8	Other specified bullous disorders
M144700	Wildfire pemphigus	L13.9	Bullous disorder, unspecified
M144800	Drug-induced pemphigus		
M145100	Benign pemphigus NOS		

Continued

For peer review only

Read code		ICD-10 code
M145200	Senile dermatitis herpetiformis	
M145300	Acquired epidermolysis bullosa	
M146.00	Benign mucous membrane pemphigoid	
M146000	Benign mucous membrane pemphigoid with no eye involvement	
M146011	Cicatricial pemphigoid	
M146100	Ocular pemphigoid	
M146z00	Benign mucous membrane pemphigoid NOS	
M14y.00	Other specified bullous dermatoses	
M14z.00	Bullous dermatoses NOS	
M151.00	Erythema multiforme	
M151.12	Toxic epidermal necrolysis	
M151700	Stevens-Johnson syndrome	
M151800	Toxic epidermal necrolysis	
M151y00	Other specified erythema multiforme	
M151z00	Erythema multiforme NOS	
M252100	Pompholyx unspecified	
M252200	Cheiropompholyx	
M252300	Podopompholyx	
M2y9000	Bullous cutaneous amyloidosis	
Myu1.00	[X]Bullous disorders	
Myu1000	[X]Other pemphigus	
Myu1200	[X]Other pemphigoid	
Myu1300	[X]Other specified bullous disorders	
Myu4100	[X]Other erythema multiforme	
PH33111	Benign familial chronic pemphigus	
PH3y200	Epidermolysis bullosa	
PH3y700	Epidermolysis bullosa simplex	
PH3y800	Epidermolysis bullosa letalis	
PH3y900	Epidermolysis bullosa dystrophica	
Pyu9100	[X]Other epidermolysis bullosa	
M2y9000	Bullous cutaneous amyloidosis	
Myu1.00	[X]Bullous disorders	

Continued

For peer review only

Read code		ICD-10 code
Myu1000	[X]Other pemphigus	
Myu1200	[X]Other pemphigoid	
Myu1300	[X]Other specified bullous disorders	
Myu4100	[X]Other erythema multiforme	
PH33111	Benign familial chronic pemphigus	
PH3y200	Epidermolysis bullosa	
PH3y700	Epidermolysis bullosa simplex	
PH3y800	Epidermolysis bullosa letalis	
PH3y900	Epidermolysis bullosa dystrophica	
Pyu9100	[X]Other epidermolysis bullosa	

For peer review only

Supplementary Table 2

Supplementary Table 2. Blistering disease codes recorded as primary or secondary diagnoses in HES for the ten patients where the algorithm generated an uncertain diagnosis

**Blistering disease codes in HES**
**Specific + specific codes**
Bullous pemphigoid (L12.0) + pemphigus vulgaris (L10.0)
Bullous pemphigoid (L12.0) + cicatricial pemphigoid (L12.1)
**Specific + broad codes**
Bullous pemphigoid (L12.0) + pemphigus unspecified (L10.9)
Bullous pemphigoid (L12.0) + bullous disorder, unspecified (L13.9)
Dermatitis herpetiformis (L13.0) + bullous disorder, unspecified (L13.9)
Pemphigus vulgaris (L10.0) + other pemphigoid (L12.8)
Pemphigus vulgaris (L10.0) + pemphigus, unspecified (L10.9) + pemphigoid, unspecified (L12.9)
**Broad + broad codes**
Pemphigus, unspecified (L10.0) + pemphigoid, unspecified (L12.9)

For peer review only

STROBE Statement—checklist of items that should be included in reports of observational studies

	Item No	Recommendation	Page
**Title and abstract**	1	(*a*) Indicate the study's design with a commonly used term in the title or the abstract	1
		(*b*) Provide in the abstract an informative and balanced summary of what was done and what was found	3
**Introduction**			
Background/rationale	2	Explain the scientific background and rationale for the investigation being reported	4-5
Objectives	3	State specific objectives, including any prespecified hypotheses	5
**Methods**			
Study design	4	Present key elements of study design early in the paper	5
Setting	5	Describe the setting, locations, and relevant dates, including periods of recruitment, exposure, follow-up, and data collection	5-7
Participants	6	(*a*) *Cohort study*—Give the eligibility criteria, and the sources and methods of selection of participants. Describe methods of follow-up<br>*Case-control study*—Give the eligibility criteria, and the sources and methods of case ascertainment and control selection. Give the rationale for the choice of cases and controls<br>*Cross-sectional study*—Give the eligibility criteria, and the sources and methods of selection of participants	6
		(*b*) *Cohort study*—For matched studies, give matching criteria and number of exposed and unexposed<br>*Case-control study*—For matched studies, give matching criteria and the number of controls per case	NA
Variables	7	Clearly define all outcomes, exposures, predictors, potential confounders, and effect modifiers. Give diagnostic criteria, if applicable	7, Figs1-2
Data sources/ measurement	8*	For each variable of interest, give sources of data and details of methods of assessment (measurement). Describe comparability of assessment methods if there is more than one group	6-7
Bias	9	Describe any efforts to address potential sources of bias	NA
Study size	10	Explain how the study size was arrived at	NA
Quantitative variables	11	Explain how quantitative variables were handled in the analyses. If applicable, describe which groupings were chosen and why	8
Statistical methods	12	(*a*) Describe all statistical methods, including those used to control for confounding	8
		(*b*) Describe any methods used to examine subgroups and interactions	NA
		(*c*) Explain how missing data were addressed	NA
		(*d*) *Cohort study*—If applicable, explain how loss to follow-up was addressed<br>*Case-control study*—If applicable, explain how matching of cases and controls was addressed<br>*Cross-sectional study*—If applicable, describe analytical methods taking account of sampling strategy	NA
		(*e*) Describe any sensitivity analyses	8

For peer review only - http://bmjopen.bmj.com/site/about/guidelines.xhtml

Continued on next page

**Results**			**Page**
Participants	13*	(a) Report numbers of individuals at each stage of study—eg numbers potentially eligible, examined for eligibility, confirmed eligible, included in the study, completing follow-up, and analysed	8
		(b) Give reasons for non-participation at each stage	NA
		(c) Consider use of a flow diagram	NA
Descriptive data	14*	(a) Give characteristics of study participants (eg demographic, clinical, social) and information on exposures and potential confounders	8-9, Fig3
		(b) Indicate number of participants with missing data for each variable of interest	NA
		(c) *Cohort study*—Summarise follow-up time (eg, average and total amount)	NA
Outcome data	15*	*Cohort study*—Report numbers of outcome events or summary measures over time	*9*
		Case-control study—Report numbers in each exposure category, or summary measures of exposure	*NA*
		Cross-sectional study—Report numbers of outcome events or summary measures	*9*
Main results	16	(*a*) Give unadjusted estimates and, if applicable, confounder-adjusted estimates and their precision (eg, 95% confidence interval). Make clear which confounders were adjusted for and why they were included	9-11
		(*b*) Report category boundaries when continuous variables were categorized	NA
		(*c*) If relevant, consider translating estimates of relative risk into absolute risk for a meaningful time period	NA
Other analyses	17	Report other analyses done—eg analyses of subgroups and interactions, and sensitivity analyses	10
**Discussion**			
Key results	18	Summarise key results with reference to study objectives	10-11
Limitations	19	Discuss limitations of the study, taking into account sources of potential bias or imprecision. Discuss both direction and magnitude of any potential bias	13-14
Interpretation	20	Give a cautious overall interpretation of results considering objectives, limitations, multiplicity of analyses, results from similar studies, and other relevant evidence	14-15
Generalisability	21	Discuss the generalisability (external validity) of the study results	14
**Other information**			
Funding	22	Give the source of funding and the role of the funders for the present study and, if applicable, for the original study on which the present article is based	2

*Give information separately for cases and controls in case-control studies and, if applicable, for exposed and unexposed groups in cohort and cross-sectional studies.

Note: An Explanation and Elaboration article discusses each checklist item and gives methodological background and published examples of transparent reporting. The STROBE checklist is best used in conjunction with this article (freely available on the Web sites of PLoS Medicine at http://www.plosmedicine.org/, Annals of Internal Medicine at http://www.annals.org/, and Epidemiology at http://www.epidem.com/). Information on the STROBE Initiative is available at www.strobe-statement.org.

For peer review only - http://bmjopen.bmj.com/site/about/guidelines.xhtml

BMJ Open

A validation study of bullous pemphigoid and pemphigus vulgaris recording in routinely collected electronic primary healthcare records in England

Journal:	*BMJ Open*
Manuscript ID	bmjopen-2019-035934.R1
Article Type:	Original research
Date Submitted by the Author:	12-Mar-2020
Complete List of Authors:	Persson, Monica; University of Nottingham Faculty of Medicine and Health Sciences, CEBD<br>Harman, Karen; University of Nottingham, Centre of Evidence Based Medicine<br>Vinogradova, Yana; University of Nottingham, Primary Care<br>Langan, Sinead; London School of Hygiene and Tropical Medicine, Department of Non-communicable Disease Epidemiology<br>Hippisley-Cox, Julia; University of Oxford, Nuffield Department of Primary Care Sciences<br>Thomas, Kim; University of Nottingham, Dermatology<br>Gran, Sonia; University of Nottingham, Centre of Evidence Based Dermatology
**Primary Subject Heading**:	Dermatology
Secondary Subject Heading:	Epidemiology
Keywords:	Dermatological epidemiology < DERMATOLOGY, DERMATOLOGY, EPIDEMIOLOGY

SCHOLARONE™
Manuscripts

I, the Submitting Author has the right to grant and does grant on behalf of all authors of the Work (as defined in the below author licence), an exclusive licence and/or a non-exclusive licence for contributions from authors who are: i) UK Crown employees; ii) where BMJ has agreed a CC-BY licence shall apply, and/or iii) in accordance with the terms applicable for US Federal Government officers or employees acting as part of their official duties; on a worldwide, perpetual, irrevocable, royalty-free basis to BMJ Publishing Group Ltd ("BMJ") its licensees and where the relevant Journal is co-owned by BMJ to the co-owners of the Journal, to publish the Work in this journal and any other BMJ products and to exploit all rights, as set out in our licence.

The Submitting Author accepts and understands that any supply made under these terms is made by BMJ to the Submitting Author unless you are acting as an employee on behalf of your employer or a postgraduate student of an affiliated institution which is paying any applicable article publishing charge ("APC") for Open Access articles. Where the Submitting Author wishes to make the Work available on an Open Access basis (and intends to pay the relevant APC), the terms of reuse of such Open Access shall be governed by a Creative Commons licence – details of these licences and which Creative Commons licence will apply to this Work are set out in our licence referred to above.

Other than as permitted in any relevant BMJ Author's Self Archiving Policies, I confirm this Work has not been accepted for publication elsewhere, is not being considered for publication elsewhere and does not duplicate material already published. I confirm all authors consent to publication of this Work and authorise the granting of this licence.

For peer review only

TITLE PAGE

Title

A validation study of bullous pemphigoid and pemphigus vulgaris recording in routinely collected electronic primary healthcare records in England

Manuscript word, table, and figure counts

Word count: 3,171

Table count: 2

Figure count: 3

Names of authors, institutions

MSM Persson[1], KE Harman[1], Y Vinogradova[2], SM Langan[3], J Hippisley-Cox[4], KS Thomas[1], S Gran[1]

[1] Centre of Evidence Based Dermatology, University of Nottingham

[2] Division of Primary Care, University of Nottingham

[3] Department of Non-communicable Disease Epidemiology, London School of Hygiene and Tropical Medicine

[4] Nuffield Department of Primary Care Health Sciences, University of Oxford

Corresponding author

Monica SM Persson,

Centre of Evidence Based Dermatology, University Of Nottingham
King's Meadow Campus, Lenton Lane,
Nottingham
NG7 2NR
UK
monica.persson1@nottingham.ac.uk

Funding sources

This work was supported by the National Institute for Health Research (NIHR) Research for Patient Benefit grant (PB-PG-0817-20033). The views expressed are those of the authors and not necessarily those of the NIHR or the Department of Health and Social

For peer review only - http://bmjopen.bmj.com/site/about/guidelines.xhtml

Care. SML reports grants from Wellcome Senior Clinical Fellowship in Science (205039/Z/16/Z) during the conduct of the study.

Competing interests

MSM Persson, KE Harman, Y Vinogradova, SM Langan, J Hippisley-Cox, KS Thomas, S Gran declare no conflict of interest.

Keywords
Bullous pemphigoid, Pemphigus vulgaris, Validation, Read code, Electronic healthcare records

Data availability

Data can be requested from www.cprd.com

For peer review only - http://bmjopen.bmj.com/site/about/guidelines.xhtml

For peer review only

ABSTRACT

Objectives

The validity of bullous pemphigoid and pemphigus vulgaris recording in routinely collected healthcare data in the UK is unknown. We assessed the positive predictive value (PPV) for bullous pemphigoid and pemphigus vulgaris primary care Read codes in the Clinical Practice Research Datalink (CPRD) using linked inpatient data (Hospital Episode Statistics; HES) as the diagnostic benchmark.

Setting

Adult participants with bullous pemphigoid or pemphigus vulgaris registered with HES-linked general practices in England between January 1998 to December 2017. Code-based algorithms were used to identify patients from the CPRD and extract their benchmark blistering disease diagnosis from HES.

Primary outcome measure

The PPVs of Read codes for bullous pemphigoid and pemphigus vulgaris.

Results

Of 2,468 incident cases of bullous pemphigoid and 431 of pemphigus vulgaris, 797 (32.3%) and 85 (19.7%) patients, respectively, had a hospitalisation record for a blistering disease. The PPV for bullous pemphigoid Read codes was 93.2% (95%CI 91.3 to 94.8). Of the bullous pemphigoid cases, 3.0% had a HES diagnosis of pemphigus vulgaris and 3.8% of another blistering disease. The PPV for pemphigus vulgaris Read codes was 58.5% (95%CI 48.0 to 68.9%). Of the pemphigus vulgaris cases, 24.7% had a HES diagnosis of bullous pemphigoid and 16.5% of another blistering disease.

Conclusions

The CPRD can be used to study bullous pemphigoid, but recording of pemphigus vulgaris needs to improve in primary care.

ARTICLE SUMMARY - STRENGTHS AND LIMITATIONS OF THIS STUDY

- This is the first study to validate the recording of bullous pemphigoid and pemphigus vulgaris in primary healthcare records in England

For peer review only - http://bmjopen.bmj.com/site/about/guidelines.xhtml

- The study involved a large sample size and the application of algorithms to identify both primary care and benchmark hospital inpatient diagnoses
- Blistering diagnoses recorded in hospital inpatient records were regarded as the benchmark diagnosis, but may be subject to misclassification

MAIN TEXT

INTRODUCTION

Bullous pemphigoid and pemphigus vulgaris are rare autoimmune blistering diseases characterised by blistering of the skin and mucous membrane and associated with high mortality.[1,2] There is a continued need to understand the causes, natural history, and disease associations in order to better inform patients and plan treatment. However, there is a scarcity of recent epidemiological research on these distinct diseases in the UK and many unanswered questions. Furthermore, interpretation of available evidence is limited by the poor external validity and lack of power that are a feature of hospital-based studies.[3-6]

Routinely collected electronic healthcare records (EHRs) may offer an excellent opportunity to examine bullous pemphigoid and pemphigus vulgaris at population-level due to the availability of data on a large number of patients that are broadly representative of the UK population.[7] Furthermore, the presence of prescription data allows the important association between possible drug exposure and bullous pemphigoid to be established, and longitudinal follow-up allows long-term outcomes to be assessed.[8] However, as EHRs were created for clinical purposes, and not directly for research, it is essential to establish the validity of the diagnoses of interest.[9]

Diagnoses of bullous pemphigoid or pemphigus vulgaris are usually made in secondary care and entered into the primary care records by general practitioners (GPs) or other practice staff using Read codes. Read codes have been used previously to identify patients with bullous pemphigoid and pemphigus vulgaris in EHRs, but have not been validated [2,8,10,11]. A previous study examined the incidence of bullous pemphigoid and pemphigus vulgaris in the UK and found that the average age of patients with

For peer review only - http://bmjopen.bmj.com/site/about/guidelines.xhtml

pemphigus vulgaris was older than observed in other studies.[2] Furthermore it coincided with the peak age for bullous pemphigoid, suggesting that pemphigus vulgaris Read codes may not be accurate and that, in some cases, bullous pemphigoid was misclassified as pemphigus vulgaris.

Due to the linkage between primary and secondary care data, we now have the opportunity to externally validate bullous pemphigoid and pemphigus vulgaris Read codes. This study aimed to determine the positive predictive value (PPV) for bullous pemphigoid and pemphigus vulgaris primary care diagnostic Read codes in Clinical Practice Research Datalink (CPRD) using linked inpatient data (Hospital Episode Statistics; HES) as the benchmark.

METHODS

Study design

This was a cohort study to validate Read codes for bullous pemphigoid and pemphigoid vulgaris in the CPRD. We followed guidelines for the reporting of studies conducted using observational routinely collected health data.[12]

Data sources

CPRD

The CPRD GOLD is a longitudinal database of UK general practices using the Vision software system. It contains anonymised healthcare records from 1987 onwards for approximately 17 million patients with a current coverage of approximately 2.7 million (4%) of the UK population. Routinely collected data within the CPRD include demographic and clinical information.[7] Symptoms and diagnoses, generated from primary care consultations or obtained from hospital discharge or specialist clinic letters, are entered by GPs or other practice staff using Read codes.[13]

The data in the CPRD have repeatedly been shown to be of good research quality.[14] At a practice-level, participating practices are assigned an “up-to-standard” date on completion of regular audits confirming data quality. At the patient-level, records are

For peer review only - http://bmjopen.bmj.com/site/about/guidelines.xhtml

assessed and patients are deemed "acceptable" if data checks indicate that their record meets pre-specified quality standards.

Hospital Episode Statistics (HES)

HES admitted patient care data are available for approximately 75% of English practices, covering over 10 million patients, that have consented to provide patient-level information from linked resources.[15] Linkage is based on each patient's NHS number, which is unique and remains unchanged through their lifetime, along with other identifiers (e.g., gender, date of birth, postcode).[16] HES admitted patient care contains demographic and clinical data, coded using the International Classification of Diseases version 10 (ICD-10)[17,18] Each hospital episode is attributed one primary diagnosis, whilst comorbidities and other diagnoses are recorded as secondary diagnoses.

Study population

Adult men and women registered with 410 HES-linked general practices in England during the period of 1 January 1998 to 31 December 2017 were included.

Case definition of bullous pemphigoid and pemphigus vulgaris

Diagnostic Read code lists for bullous pemphigoid and pemphigus vulgaris were developed based on the expert opinion of two consultant dermatologists (KEH and SML) and a GP (JHC). Bullous pemphigoid Read codes were classed as specific (M145000, "bullous pemphigoid") or broad (M145.00, "pemphigoid"; M145z00, "pemphigoid not otherwise specified"). Likewise, pemphigus vulgaris codes were grouped as specific (M144600, "pemphigus vulgaris"; M144500, "pemphigus vegetans") or broad (M144.00, "pemphigus"; M144z00, "pemphigus not otherwise specified").

Clinical data were sought for all patients that had at least one specific or broad Read code for bullous pemphigoid or pemphigus vulgaris. Based on clinical experience, it was considered inappropriate to define a case based on the first recording of bullous pemphigoid or pemphigus vulgaris without taking into account subsequent Read code entries for blistering diseases which were sometimes multiple and conflicting. A code-based algorithm (Figure 1) was therefore applied to determine the most likely diagnosis.

For peer review only - http://bmjopen.bmj.com/site/about/guidelines.xhtml

The date of diagnosis was taken as the first recording of the Read code generated through the algorithm. Patients were considered cases if their most likely blistering disease diagnosis, generated through the algorithm, was bullous pemphigoid or pemphigus vulgaris. Those with diagnoses for other blistering diseases were excluded. Further information about the blistering disease codes used is available in Supplementary Table 1.

Observation period

The observation period commenced on the latest of (i) 1st January 1998, (ii) the patient's 18th birthday, (iii) 1 year after the patient was registered with their current general practice, or (iv) the practice's up-to-standard date. A 1-year lag period from the patient's registration date with their GP was imposed to minimise the risk of prevalent cases being identified as incident cases.[19]

The observation period terminated on the earliest of (i) 31st December 2017, (ii) the date of death, (iii) the date the patient left the practice, (iv) the practice's last data collection date, (v) the most recent linkage date between CPRD and HES.

Validation of bullous pemphigoid and pemphigus vulgaris Read codes

For people with a bullous pemphigoid or pemphigus vulgaris diagnosis in primary care, we identified those with a hospital inpatient episode recording a blistering disease (ICD-10: L10, L12, L13) as a primary or secondary diagnosis at any point. Due to the presence of multiple records per patient, an algorithm based on clinical knowledge (Figure 2) was applied to determine one secondary care ICD-10 blistering disease diagnosis per patient, which was regarded as their benchmark diagnosis. The date of diagnosis was taken as the first recording of the diagnosis generated through the algorithm. The distribution of the benchmark diagnoses was examined for bullous pemphigoid and pemphigus vulgaris cases identified in the CPRD.

ICD-10 codes L12.0 (bullous pemphigoid) and L12.9 (pemphigoid, unspecified) were classed as "bullous pemphigoid". ICD-10 codes L10.0 (pemphigus vulgaris), L10.1 (pemphigus vegetans), and L10.9 (pemphigus, unspecified) were classed as "pemphigus

For peer review only - http://bmjopen.bmj.com/site/about/guidelines.xhtml

vulgaris”. All other blistering disease ICD-10 codes and where the HES algorithm generated an uncertain diagnosis were classed as “other”. See Supplementary Table 1 for the full list of blistering disease codes.

Statistical analysis

The sex and age of bullous pemphigoid and pemphigus vulgaris cases were presented descriptively. Age was assessed for normality visually, the mean (standard deviation, SD) or median (interquartile range, IQR) were calculated as appropriate. For bullous pemphigoid and pemphigus vulgaris cases with a hospital inpatient record for a blistering disease, the PPV and associated 95% confidence interval (CI) were calculated for the specific, broad, and combined Read codes for bullous pemphigoid and pemphigus vulgaris against their benchmark diagnosis from HES. The blistering disease secondary care diagnoses were presented descriptively. Analyses were conducted using Stata 16 (StataCorp LP).

Sensitivity analysis

The most recent Read code was used for patients with conflicting Read codes (i.e., patients who did not have a more commonly occurring Read code; last step of algorithm, Figure 1). This was done under the assumption that diagnoses are likely to be refined over time and the most recent code was likely to be most accurate. The impact of this assumption was investigated through a sensitivity analysis comparing the PPVs for bullous pemphigoid and pemphigus vulgaris in all cases versus excluding patients with conflicting Read codes.

Ethical approval

The present study was approved by the Independent Scientific Advisory Committee for the CPRD (ISAC protocol no 18_224).

Patient and public involvement

The study was supported by a patient and public advisory group which provided input to the programme of research. We met with this advisory group on a regular basis for the duration of the study. The advisory group commented on the design of the study and, at

For peer review only - http://bmjopen.bmj.com/site/about/guidelines.xhtml

the end of the study, they commented on the findings and suggested how to share the findings with the public.

RESULTS

Study population

A total of 2,899 incident cases of bullous pemphigoid and pemphigus vulgaris were identified in HES-linked practices using the algorithm (see Supplementary Figure 1 for patient flow diagram). The median (IQR) age at diagnosis was 81 years (73 to 87) for the 2,468 bullous pemphigoid cases, and 64 years (48 to 77) for the 431 pemphigus vulgaris cases. The peak age for bullous pemphigoid recording was around 80-84 years. In contrast, two peaks were observed for pemphigus vulgaris case recording, one at 50-54 years and a later, slightly higher, peak at 75-79 years (Figure 3).

The proportion of women were 56.7% and 64.5% for bullous pemphigoid and pemphigus vulgaris, respectively. Of the 2,899 cases, 223 (9.0%) had at least one conflicting Read code indicative of another blistering disease or a general blistering disease code (e.g., “bullous dermatoses”).

Validation of bullous pemphigoid and pemphigus vulgaris diagnostic codes

Of 2,899 incident cases of bullous pemphigoid or pemphigus vulgaris, 882 (30.4%) had at least one hospital episode recording a blistering disease diagnosis. In 44.0% of these patients, it was a primary diagnosis. The remaining 2,017 (69.6%) patients did not have any hospitalisation records listing a blistering disease diagnosis and were not analysed in the validation study.

Using the algorithm, ten patients (1.1%) had an uncertain HES diagnosis and were classified as “other”. The blistering disease codes of these cases are shown in Supplementary Table 2.The distribution of HES diagnoses for the bullous pemphigoid and pemphigus vulgaris incident cases and PPV for bullous pemphigoid and pemphigus vulgaris Read codes are shown in Table 1.

Table 1. Distribution of benchmark blistering disease diagnoses for bullous pemphigoid and pemphigus vulgaris incident cases identified in the CPRD, shown with the PPV (95%CI) for specific, broad, and

For peer review only - http://bmjopen.bmj.com/site/about/guidelines.xhtml

combined Read codes for each disease

CPRD Read code	Hospital episode diagnosis (benchmark)				
	Bullous pemphigoid	Pemphigus vulgaris	Other	n	% PPV (95%CI)
**Bullous pemphigoid**					
Specific (M145000)	527 (95.6%)	12 (2.2%)	12 (2.2%)	551	95.6 (93.6 to 97.1)
Broad (M145.00, M145z00)	216 (87.8%)	12 (4.9%)	18 (7.3%)	246	87.8 (83.1 to 91.4)
Total	743 (93.2%)	24 (3.0%)	30 (3.8%)	797	93.2 (91.3 to 94.8)
**Pemphigus vulgaris**					
Specific (M144600, M144500)	2 (9.1%)	18 (81.8%)	2 (9.1%)	22	81.8 (58.8 to 93.4)
Broad (M144.00, M144z00)	19 (30.2%)	32 (50.8%)	12 (19.1%)	63	50.8 (38.4 to 63.1)
Total	21 (24.7%)	50 (58.8%)	14 (16.5%)	85	58.8 (48.0 to 68.9)

ICD-10 codes L10.0 (pemphigus vulgaris), L10.1 (pemphigus vegetans), and L10.9 (pemphigus, unspecified) are classed as a hospital episode diagnosis of "pemphigus vulgaris". ICD-10 codes L12.0 (bullous pemphigoid) and L12.9 (pemphigoid, unspecified) are classed as "bullous pemphigoid". All other blistering disease ICD-10 codes and where the HES algorithm generated an uncertain diagnosis (n=7 for bullous pemphigoid and n=3 for pemphigus vulgaris) are classed as "other"

Bullous pemphigoid

Of 2,468 incident cases, 797 (32.2%) had a hospitalisation record for a blistering disease. Cases were most commonly coded with specific (69.1%) rather than broad (30.8%) Read codes. The PPV of the bullous pemphigoid Read codes combined was 93.2% (95%CI 91.3 to 94.8). Broad Read codes had a lower PPV (87.8%, 95%CI 83.1 to 91.4) than the specific codes (95.6%, 95%CI 93.6 to 97.1).

Pemphigus vulgaris

Of 431 incident cases, 85 (20%) had a hospitalisation record for a blistering disease. Cases were more commonly coded with broad disease codes (74.1%) than specific codes (25.9%). The PPV of the pemphigus vulgaris Read codes combined was 58.8% (95%CI 48.0 to 68.9). Specific Read codes showed a higher PPV (81.8%, 95%CI 58.8 to 93.4) than broad codes (50.8%, 95%CI 38.4 to 63.1).

Sensitivity analysis

The sensitivity analyses excluding the 223 patients with conflicting Read codes resulted in broadly unchanged PPVs. The PPV for the 776 patients with consistent bullous

For peer review only - http://bmjopen.bmj.com/site/about/guidelines.xhtml

pemphigoid Read codes was 93.6% (95%CI 91.6 to 95.1). The PPV for the 75 patients with consistent pemphigus vulgaris Read codes was 61.3% (95%CI 49.7 to 71.8).

Inconsistent codes

Bullous pemphigoid

A benchmark, secondary care diagnosis of pemphigus vulgaris or another blistering disease diagnosis was made for 24 (3.0%) and 30 (3.8%) of the 797 cases, respectively. The most common "other" benchmark diagnosis was cicatricial pemphigoid (n=11, 36.7%; L12.1, mucous membrane pemphigoid). Other benchmark diagnoses were other pemphigus (L10.8), other pemphigoid (L12.8), and bullous disorder unspecified (L13.9).

Pemphigus vulgaris

Of the 85 cases with a primary care diagnosis for pemphigus vulgaris, 21 (24.7%) had a HES diagnosis for bullous pemphigoid and 14 (16.5%) for another blistering diagnosis. The most common "other" secondary care diagnosis was for pemphigus foliaceous (n=6, 42.9%; L10.2). Other benchmark diagnosis were drug-induced pemphigus (L10.5), cicatricial pemphigoid (L12.1), dermatitis herpetiformis (L13.0), subcorneal pustular dermatitis (L13.1), and other specified bullous disorder (L13.8).

Discussion

Main findings

Incident diagnoses of bullous pemphigoid and pemphigus vulgaris identified in the CPRD were compared to the blistering disease diagnoses made in secondary care (benchmark diagnosis). Diagnoses were recorded in the CPRD using specific (M145000, "bullous pemphigoid"; M144600, "pemphigus vulgaris"; M144500, "pemphigus vegetans") or broad (M145.00, "pemphigoid"; M145z00, "pemphigoid not otherwise specified"; M144.00, "pemphigus"; M144z00, "pemphigus not otherwise specified") Read codes. Overall, 882 (30.4%) patients identified in the CPRD also had hospitalisation records for a blistering disease. We found good consistency between primary and secondary care diagnoses for bullous pemphigoid and a high PPV for bullous pemphigoid Read codes (93.2%). The PPV for the specific Read codes for bullous pemphigoid was higher

For peer review only - http://bmjopen.bmj.com/site/about/guidelines.xhtml

(95.6%) than that of the broad Read codes (87.8%) and captured a greater proportion of cases (69.1%). The consistency was not as good for pemphigus vulgaris, with a moderate PPV for pemphigus vulgaris Read codes (58.8%). The majority of cases of pemphigus vulgaris were coded with broad disease codes (74.1%) with lower PPVs (50.8%). The PPV for the specific pemphigus vulgaris Read codes was higher (81.8%), but captured only a subset of the population (25.9%).

Our study shows that the codes for bullous pemphigoid in the CPRD appear to be capturing people who have the disease and cases reflect expected age distributions. Approximately 93% of bullous pemphigoid cases identified using the algorithm are likely to have bullous pemphigoid. In contrast, only 59% of pemphigus vulgaris cases identified are likely to have pemphigus vulgaris, with almost 25% probably suffering from bullous pemphigoid. The probable misclassification of bullous pemphigoid cases as pemphigus vulgaris likely explains the peak of pemphigus vulgaris codes observed at 75-79 years, coinciding with the peak age for bullous pemphigoid. Consequently, the use of data from the CPRD for pemphigus vulgaris research may be limited.

Comparability with other studies

To date, no studies have validated Read codes and only two studies have validated ICD codes for bullous pemphigoid or pemphigus vulgaris using EHRs (Table 2). The PPV for bullous pemphigoid Read codes in the CPRD is comparable to that found in routinely collected healthcare data from Sweden (National Patient Register; 92%), which validated bullous pemphigoid ICD-10 codes using information from pathology registers and medical records.[20] In contrast, the PPVs for bullous pemphigoid and pemphigus vulgaris Read codes were lower in the CPRD than those presented for the electronic medical records in the US (Northwestern Medicine Electronic Data Warehouse; 99% for bullous pemphigoid and 100% for pemphigus ICD-9 codes)[21]. However, Hsu et al[21] presented the PPV only for those cases with multiple disease codes for the same disease, and not for those with a single code or those with conflicting codes. The stringency of the case definition may explain the higher PPV found, but would not be suitable if the study aim

For peer review only - http://bmjopen.bmj.com/site/about/guidelines.xhtml

was to identify all (or nearly all) cases in a population-based study. Furthermore, the work included several subsets of pemphigus disease, rather than just pemphigus vulgaris, and did not provide any indication of potential misclassification between different pemphigus diseases. Further comparisons between our study and the aforementioned studies are hindered as the studies validated ICD-9 and ICD-10 codes rather than Read codes. However, the evidence indicates that routinely collected healthcare data may be a valuable source for investigating bullous pemphigoid. The role of such data sources for investigating pemphigus vulgaris remains uncertain.

For peer review only

Table 2. Published validation studies of bullous pemphigoid or pemphigus vulgaris diagnostic codes in EHR

Publication	Disease	Classification	EHR	Population	Validation method	PPV (95%CI)
**Hsu 2016**	Pemphigus* (694.4) and bullous pemphigoid (694.5)	ICD-9 Clinical Modification diagnostic codes	Northwestern Medicine Electronic Data Warehouse; Inpatient + outpatient data	Patients with multiple codes for either pemphigus (n=161) or bullous pemphigoid (n=126).	Manual review of outpatient and inpatient records. Diagnosis was confirmed by ≥2 indicators of disease:<br>• Clinical diagnosis<br>• Biopsy histology<br>• Direct immunofluorescence<br>• Indirect immunoflourescence or enzyme-linked immunosorbent assay.	Pemphigus: 100% (96 to 100); Bullous pemphigoid: 99% (93 to 99)
**Grönhagen 2017**	Bullous pemphigoid (L12.0, L12.8, L12.9)	ICD-10 diagnostic codes	Swedish National Patient Register; Inpatient + outpatient data	307 patients with primary or secondary diagnosis of bullous pemphigoid who had medical records available for review	Manual review of immunopathological and histopathological registries and medical records. Diagnosis was confirmed by:<br>• Clear clinical history and physical examination, and<br>• Positive enzyme-linked immunosorbent assay, or<br>• Histopathological or immunopathological pattern consistent with bullous pemphigoid	92%

* Pemphigus vulgaris, pemphigus vegetans, pemphigus foliaceus, pemphigus erythematosus, paraneoplastic pemphigus, and drug induced pemphigus

Strengths and limitations

The strengths of the present work include a large sample size and the application of algorithms to identify both primary care and benchmark hospital inpatient diagnoses. One-off occurrences of a bullous pemphigoid or pemphigus vulgaris code in the presence of alternate blistering disease codes may indicate a working diagnosis that is later revised or may be incorrect due to a typographical error or oversight. Based on clinical knowledge, using an algorithm that prioritised diagnoses with multiple entries and consistent coding was considered to help reduce the impact of recording errors.

The study is limited by the inability to calculate the negative predictive value, sensitivity, and specificity of the Read codes. Additionally, HES diagnoses were regarded as the

For peer review only

For peer review only - http://bmjopen.bmj.com/site/about/guidelines.xhtml

benchmark in the present study, but may be inaccurate. The benchmark diagnoses may be subject to misclassification bias due to a heterogeneous group of pemphigoid variants (e.g., non-bullous pemphigoid, which presents with intense itching, a polymorphic rash, but no blisters), alternative approaches to making a diagnosis, and differences in the diagnostic tests used to confirm the diagnosis.[22,23] Nevertheless, this was regarded as the best approach as external validation with hospital notes was not possible. Finally, validation was only possible for a third of patients and it may be that patients with a hospitalisation record for a blistering disease are not representative of the overall population of patients as they are more likely to have severe disease and associated comorbidities.

Possible explanations for findings

Almost a quarter of patients with Read codes indicative of pemphigus vulgaris in the CPRD may have bullous pemphigoid. Such misclassification may be due to unfamiliarity with the disease by GPs and administrators or typographical error. Pemphigus and pemphigoid sound similar and, to those unfamiliar with the diseases, the terms may be thought to be interchangeable. Additionally, the lower prevalence of pemphigus vulgaris in the population might explain why lower PPVs were reported for pemphigus vulgaris than bullous pemphigoid.

Inclusion of broad Read codes also contribute to the apparent misclassification seen. For example, although we have assumed that broad pemphigoid codes identify a bullous pemphigoid patient such a code could correctly be attributed to a patient with a different pemphigoid disease, such as mucous membrane pemphigoid. Similarly, a patient with pemphigus foliaceous could correctly be attributed a broad pemphigus code. Correct attribution of broad Read codes to patients with pemphigoid or pemphigus diseases other than bullous pemphigoid or pemphigus vulgaris likely explain the lower PPVs seen for the broad versus specific Read codes.

For peer review only - http://bmjopen.bmj.com/site/about/guidelines.xhtml

Possible implications for future research

Implementation of an algorithm allows a consistent and reproducible approach to identifying patients in the presence of multiple, sometimes conflicting, blistering disease codes. The algorithm was developed based on clinical expertise and future research should be conducted to test alternate approaches and determine the sensitivity, specificity, and negative predictive value.

Focused efforts on increasing GPs' awareness of autoimmune blistering skin disorders and providing resources for how to differentiate between bullous pemphigoid and pemphigus vulgaris may be required to improve the recording of the diseases in the CPRD. Currently, the discrepant codes observed complicates the examination of pemphigus vulgaris in the CPRD, and may also underestimate the true incidence of bullous pemphigoid. However, only a minor proportion of the population with a benchmark diagnosis for bullous pemphigoid (<3%) were miscoded as pemphigus vulgaris in primary care, and the effect may therefore be minimal.

Conclusion

The PPV of Read codes for bullous pemphigoid are high in the CPRD, but only moderate for pemphigus vulgaris. The CPRD is a useful data source for bullous pemphigoid research, but may have lower utility for pemphigus vulgaris. We suggest future studies of bullous pemphigoid in the CPRD should consider using this study's validated Read code list to identify cases. EHRs, such as the CPRD, could be an invaluable resource for the examination of a rare disease such as pemphigus vulgaris, but there is a need for more accurate recording of diagnoses in primary care.

Acknowledgments

This study is based in part on data from the Clinical Practice Research Datalink obtained under licence from the UK Medicines and Healthcare products Regulatory Agency. The data is provided by patients and collected by the NHS as part of their care and support. HES data: Copyright © 2019, re-used with the permission of The Health & Social Care

For peer review only - http://bmjopen.bmj.com/site/about/guidelines.xhtml

Information Centre. All rights reserved. The interpretation and conclusions contained in this study are those of the authors alone.

Author contributions

MSMP, KEH, YV, SML, JHC, KST, and SG contributed to the conception and design. MSMP, YV, and SG contributed to data acquisition and analysis. MSMP, KEH, YV, SML, JHC, KST, and SG contributed to the interpretation of the data. MSMP drafted the manuscript and KEH, YV, SML, JHC, KST, and SG critically revised the manuscript. SG is the guarantor of the work.

References

1 Kridin K, Schwartz N, Cohen AD *et al.* Mortality in bullous pemphigoid: A systematic review and meta-analysis of standardized mortality ratios. *The Journal of Dermatology* 2018; **45**: 1094-100.

2 Langan S, Smeeth L, Hubbard R *et al.* Bullous pemphigoid and pemphigus vulgaris—incidence and mortality in the UK: population based cohort study. *Bmj* 2008; **337**: a180.

3 Gudi VS, White MI, Cruickshank N *et al.* Annual incidence and mortality of bullous pemphigoid in the Grampian Region of North-east Scotland. *Br J Dermatol* 2005; **153**: 424-7.

4 Lloyd-Lavery A, Chi C-C, Wojnarowska F *et al.* The Associations Between Bullous Pemphigoid and Drug Use: A UK Case-Control Study. *JAMA Dermatology* 2013; **149**: 58-62.

5 Taghipour K, Chi C-C, Vincent A *et al.* The Association of Bullous Pemphigoid With Cerebrovascular Disease and Dementia: A Case-Control Study. *JAMA Dermatology* 2010; **146**: 1251-4.

6 Saha M, Bhogal B, Black MM *et al.* Prognostic factors in pemphigus vulgaris and pemphigus foliaceus. *Br J Dermatol* 2014; **170**: 116-22.

7 Herrett E, Gallagher AM, Bhaskaran K *et al.* Data resource profile: clinical practice research datalink (CPRD). *International journal of epidemiology* 2015; **44**: 827-36.

8 Douros A, Rouette J, Yin H *et al.* Dipeptidyl Peptidase-4 Inhibitors and the Risk of Bullous Pemphigoid Among Patients With Type 2 Diabetes. *Diabetes care* 2019: dc190409.

9 Manuel DG, Rosella LC, Stukel TA. Importance of accurately identifying disease in studies using electronic health records. *BMJ* 2010; **341**: c4226.

10 Langan SM, Hubbard R, Fleming K *et al.* A population-based study of acute medical conditions associated with bullous pemphigoid. *British Journal of Dermatology* 2009; **161**: 1149-52.

11 Langan SM, Groves RW, West J. The relationship between neurological disease and bullous pemphigoid: a population-based case–control study. *Journal of Investigative Dermatology* 2011; **131**: 631-6.

12 Benchimol EI, Manuel DG, To T *et al.* Development and use of reporting guidelines for assessing the quality of validation studies of health administrative data. *Journal of Clinical Epidemiology* 2011; **64**: 821-9.

13 Chisholm J. The Read clinical classification. *BMJ: British Medical Journal* 1990; **300**: 1092.

For peer review only

14 Herrett E, Thomas SL, Schoonen WM *et al.* Validation and validity of diagnoses in the General Practice Research Database: a systematic review. *British journal of clinical pharmacology* 2010; **69**: 4-14.

15 Datalink CPR. Release Notes: CPRD GOLD June 2019. In: (Agency MHpR, ed). 2019.

16 Padmanabhan S, Carty L, Cameron E *et al.* Approach to record linkage of primary care data from Clinical Practice Research Datalink to other health-related patient data: overview and implications. *European journal of epidemiology* 2019; **34**: 91-9.

17 Organization WH. International statistical classification of diseases and related health problems: 10th revision (ICD-10). *http://www. who. int/classifications/apps/icd/icd* 1992.

18 Herbert A, Wijlaars L, Zylbersztejn A *et al.* Data Resource Profile: Hospital Episode Statistics Admitted Patient Care (HES APC). *International Journal of Epidemiology* 2017; **46**: 1093-i.

19 Lewis JD, Bilker WB, Weinstein RB *et al.* The relationship between time since registration and measured incidence rates in the General Practice Research Database. *Pharmacoepidemiology and drug safety* 2005; **14**: 443-51.

20 Grönhagen C, Nilzen K, Seifert O *et al.* Bullous Pemphigoid: Validation of the National Patient Register in two counties in Sweden, 2001 to 2012. *Acta dermato-venereologica* 2017; **97**: 32-5.

21 Hsu D, Brieva J, Nardone B *et al.* Validation of database search strategies for the epidemiological study of pemphigus and pemphigoid. *The British journal of dermatology* 2016; **174**: 645.

22 Smith H, Mohd Mustapa M, Cheung S *et al.* National audit on the management of bullous pemphigoid. *Clinical and experimental dermatology* 2019.

23 Lamberts A, Meijer JM, Jonkman MF. Nonbullous pemphigoid: A systematic review. *Journal of the American Academy of Dermatology* 2018; **78**: 989-95. e2.

FIGURE LEGENDS

Figure 1 - The code-based algorithm used to determine a diagnosis of bullous pemphigoid or pemphigus vulgaris using Read codes in the CPRD. The date of diagnosis was taken as the first recording of the Read code generated through the algorithm

Figure 2 - Code-based algorithm used to determine a benchmark diagnosis from secondary care HES data in patients identified from CPRD as having bullous pemphigoid or pemphigus vulgaris. The date of diagnosis was taken as the first recording of the diagnosis generated through the algorithm

Figure 3 - Age at diagnosis (years) in patients with bullous pemphigoid (n=2,468) and pemphigus vulgaris (n=431). A peak is observed at 80-84 years for bullous pemphigoid

For peer review only - http://bmjopen.bmj.com/site/about/guidelines.xhtml

recording, whilst two peaks are seen for pemphigus vulgaris; one at 50-54 and a later, and slightly higher, peak at 75-79 years

For peer review only

For peer review only - http://bmjopen.bmj.com/site/about/guidelines.xhtml

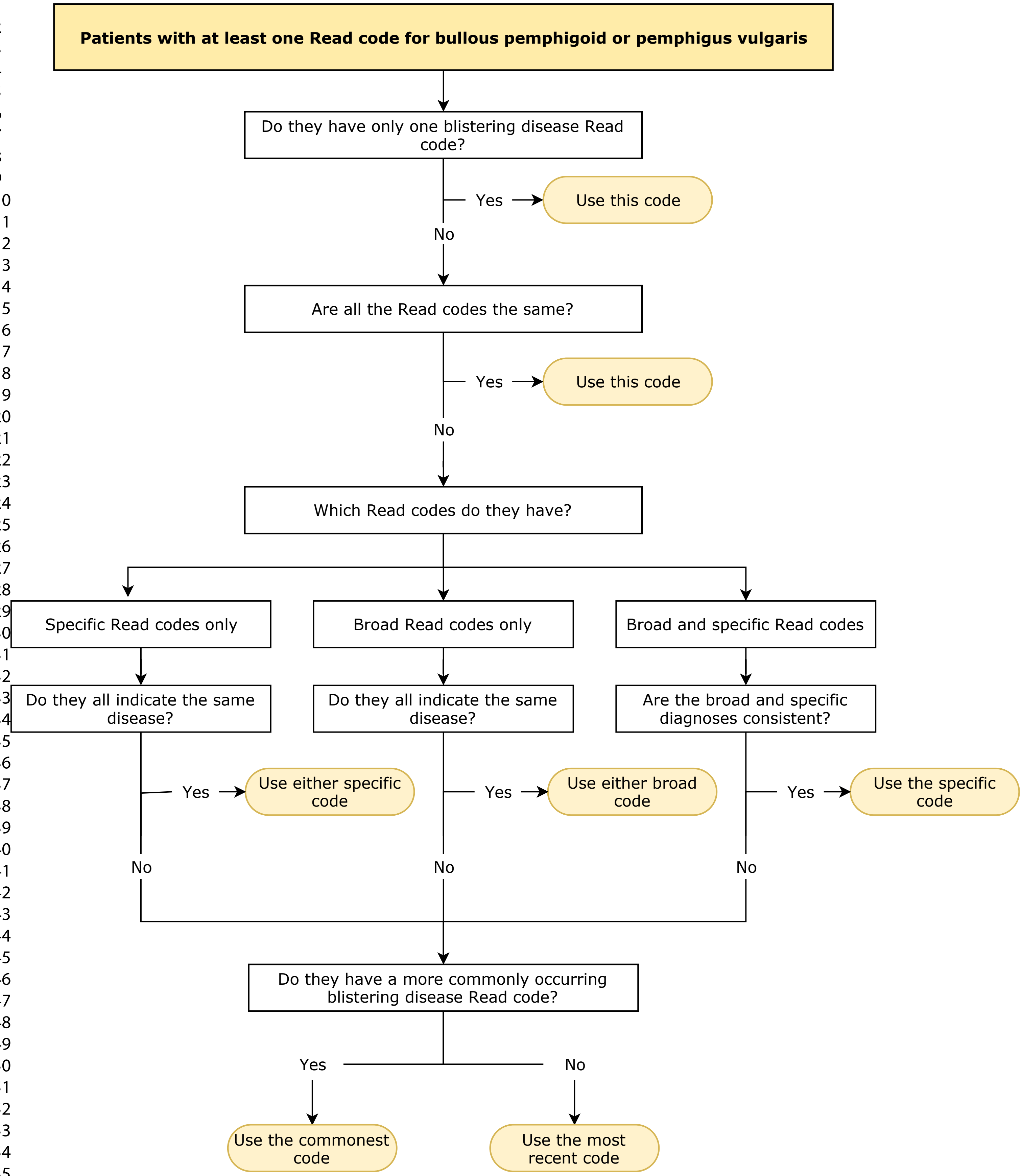
Patients with at least one Read code for bullous pemphigoid or pemphigus vulgaris
Do they have only one blistering disease Read code?
Yes
Use this code
No
Are all the Read codes the same?
Yes
Use this code
No
Which Read codes do they have?
Specific Read codes only
Broad Read codes only
Broad and specific Read codes
Do they all indicate the same disease?
Do they all indicate the same disease?
Are the broad and specific diagnoses consistent?
Yes
Use either specific code
Yes
Use either broad code
Yes
Use the specific code
No
No
No
Do they have a more commonly occurring blistering disease Read code?
Yes
No
Use the commonest code
Use the most recent code

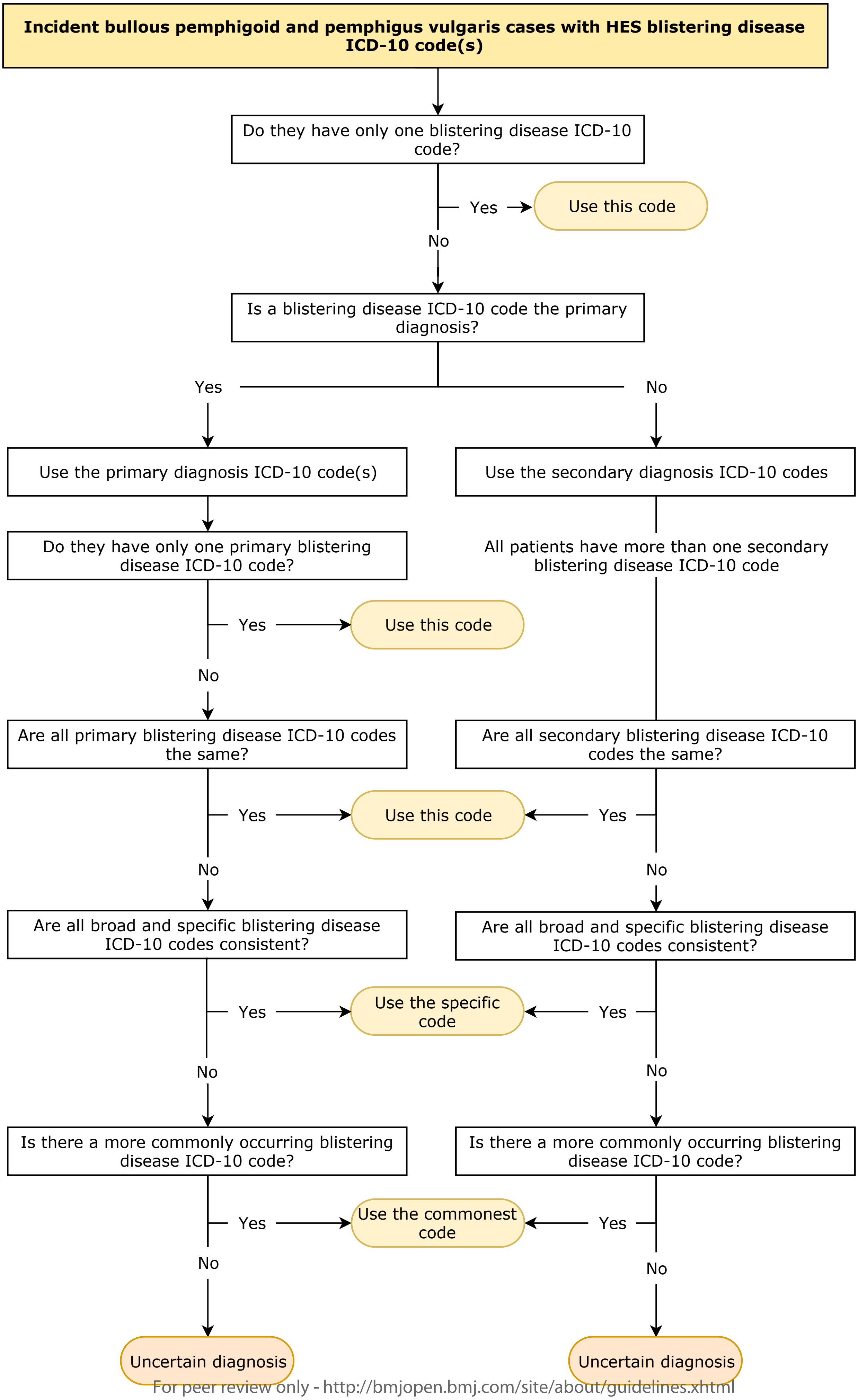
Incident bullous pemphigoid and pemphigus vulgaris cases with HES blistering disease ICD-10 code(s)
Do they have only one blistering disease ICD-10 code?
Yes
Use this code
No
Is a blistering disease ICD-10 code the primary diagnosis?
Yes
No
Use the primary diagnosis ICD-10 code(s)
Use the secondary diagnosis ICD-10 codes
Do they have only one primary blistering disease ICD-10 code?
All patients have more than one secondary blistering disease ICD-10 code
Yes
Use this code
No
Are all primary blistering disease ICD-10 codes the same?
Are all secondary blistering disease ICD-10 codes the same?
Yes
Use this code
Yes
No
No
Are all broad and specific blistering disease ICD-10 codes consistent?
Are all broad and specific blistering disease ICD-10 codes consistent?
Yes
Use the specific code
Yes
No
No
Is there a more commonly occurring blistering disease ICD-10 code?
Is there a more commonly occurring blistering disease ICD-10 code?
Yes
Use the commonest code
Yes
No
No
Uncertain diagnosis
Uncertain diagnosis

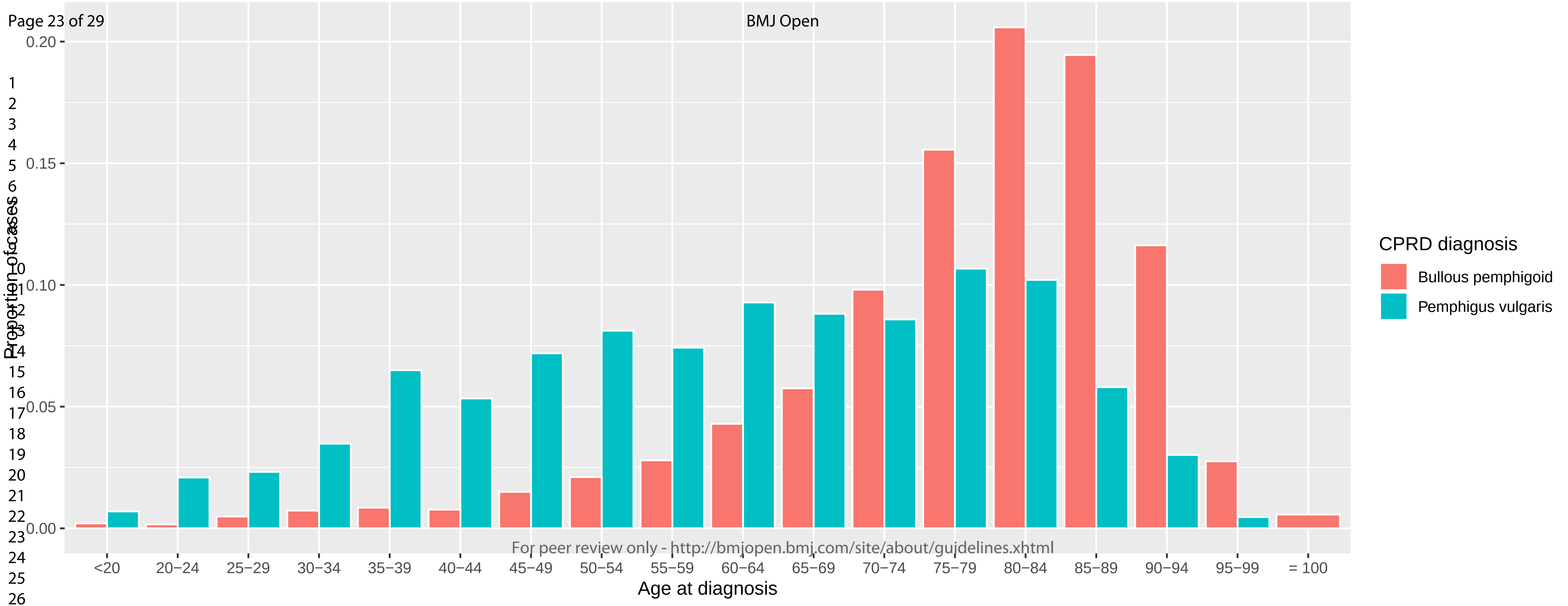

For peer review only - http://bmjopen.bmj.com/site/about/guidelines.xhtml

Supporting information

Supplementary Table 1

The CPRD and HES algorithms used to determine the most likely blistering disease diagnosis were run using all entries for the blistering diseases described below. Based on clinical expertise, we determined Read and ICD-10 codes for bullous pemphigoid and pemphigus vulgaris. We also generated a list of "other blistering diseases" which could be confused with or be differential diagnoses for bullous pemphigoid or pemphigus vulgaris.

For peer review only

Supplementary Table 1. Blistering diseases and their associated Read or ICD-10 codes according to their disease category

Read code		ICD-10 code	
**Bullous pemphigoid**			
Specific codes			
M1450	Bullous pemphigoid	L12.0	Bullous pemphigoid
Broad codes			
M145.	Pemphigoid	L12.9	Pemphigoid, unspecified
M145z	Pemphigoid NOS		
**Pemphigus vulgaris**			
Specific codes			
M1445	Pemphigus vegetans	L10.1	Pemphigus vegetans
M1446	Pemphigus vulgaris	L10.0	Pemphigus vulgaris
Broad codes			
M144.	Pemphigus	L10.9	Pemphigus, unspecified
M144z	Pemphigus NOS		
**Other blistering diseases**			
2F6..11	O/E – pompholyx	L10.2	Pemphigus foliaceous
A900.17	Congenital syphilitic pemphigus	L10.3	Brazilian pemphigus
C371400	Porphyria cutanea tarda	L10.4	Pemphigus erythematosus
F4Cy100	Ocular pemphigoid	L10.5	Drug-induced pemphigus
M14..00	Bullous dermatoses	L10.8	Other pemphigus
M140.00	Dermatitis herpetiformis	L12.1	Cicatricial pemphigoid
M141.00	Subcorneal pustular dermatosis	L12.2	Chronic bullous disease of childhood

Continued

Read code		ICD-10 code	
M142.00	Juvenile dermatitis herpetiformis	L12.3	Acquired epidermolysis bullosa
M142.11	Juvenile pemphigoid	L12.8	Other pemphigoid
M144000	Benign pemphigus	L13.0	Dermatitis herpetiformis
M144200	Erythematous pemphigus	L13.1	Subcorneal pustular dermatitis
M144300	Foliaceous pemphigus	L13.8	Other specified bullous disorders
M144700	Wildfire pemphigus	L13.9	Bullous disorder, unspecified
M144800	Drug-induced pemphigus		
M145100	Benign pemphigus NOS		
M145200	Senile dermatitis herpetiformis		
M145300	Acquired epidermolysis bullosa		
M146.00	Benign mucous membrane pemphigoid		
M146000	Benign mucous membrane pemphigoid with no eye involvement		
M146011	Cicatricial pemphigoid		
M146100	Ocular pemphigoid		
M146z00	Benign mucous membrane pemphigoid NOS		
M14y.00	Other specified bullous dermatoses		
M14z.00	Bullous dermatoses NOS		
M151.00	Erythema multiforme		
M151.12	Toxic epidermal necrolysis		
M151700	Stevens-Johnson syndrome		
M151800	Toxic epidermal necrolysis		
M151y00	Other specified erythema multiforme		
M151z00	Erythema multiforme NOS		
M252100	Pompholyx unspecified		
M252200	Cheiropompholyx		
M252300	Podopompholyx		
M2y9000	Bullous cutaneous amyloidosis		
Myu1.00	[X]Bullous disorders		
Myu1000	[X]Other pemphigus		
Myu1200	[X]Other pemphigoid		
Myu1300	[X]Other specified bullous disorders		
Myu4100	[X]Other erythema multiforme		

Continued

For peer review only

Read code		ICD-10 code
PH33111	Benign familial chronic pemphigus	
PH3y200	Epidermolysis bullosa	
PH3y700	Epidermolysis bullosa simplex	
PH3y800	Epidermolysis bullosa letalis	
PH3y900	Epidermolysis bullosa dystrophica	
Pyu9100	[X]Other epidermolysis bullosa	
M2y9000	Bullous cutaneous amyloidosis	
Myu1.00	[X]Bullous disorders	
Myu1000	[X]Other pemphigus	
Myu1200	[X]Other pemphigoid	
Myu1300	[X]Other specified bullous disorders	
Myu4100	[X]Other erythema multiforme	
PH33111	Benign familial chronic pemphigus	
PH3y200	Epidermolysis bullosa	
PH3y700	Epidermolysis bullosa simplex	
PH3y800	Epidermolysis bullosa letalis	
PH3y900	Epidermolysis bullosa dystrophica	
Pyu9100	[X]Other epidermolysis bullosa	

For peer review only

Supplementary Figure 1

Flow diagram showing identification of incident cases of bullous pemphigoid and pemphigus vulgaris for inclusion in the validation study

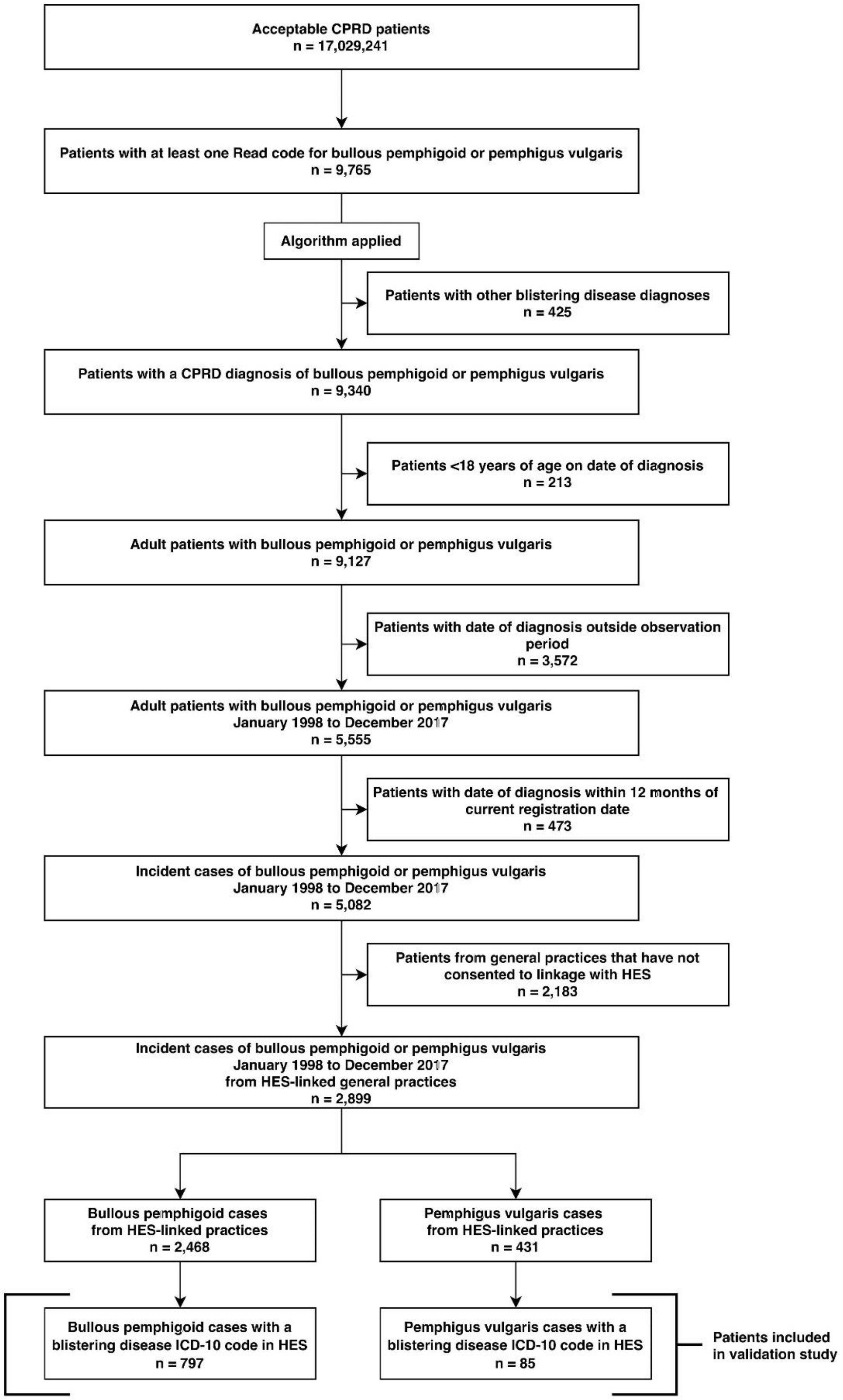

Supplementary Table 2

Supplementary Table 2. Blistering disease codes recorded as primary or secondary diagnoses in HES for the ten patients where the algorithm generated an uncertain diagnosis

**Blistering disease codes in HES**
**Specific + specific codes**
Bullous pemphigoid (L12.0) + pemphigus vulgaris (L10.0)
Bullous pemphigoid (L12.0) + cicatricial pemphigoid (L12.1)
**Specific + broad codes**
Bullous pemphigoid (L12.0) + pemphigus unspecified (L10.9)
Bullous pemphigoid (L12.0) + bullous disorder, unspecified (L13.9)
Dermatitis herpetiformis (L13.0) + bullous disorder, unspecified (L13.9)
Pemphigus vulgaris (L10.0) + other pemphigoid (L12.8)
Pemphigus vulgaris (L10.0) + pemphigus, unspecified (L10.9) + pemphigoid, unspecified (L12.9)
**Broad + broad codes**
Pemphigus, unspecified (L10.0) + pemphigoid, unspecified (L12.9)

For peer review only

STROBE Statement—checklist of items that should be included in reports of observational studies

	Item No	Recommendation	Page
**Title and abstract**	1	(*a*) Indicate the study's design with a commonly used term in the title or the abstract	1
		(*b*) Provide in the abstract an informative and balanced summary of what was done and what was found	3
**Introduction**			
Background/rationale	2	Explain the scientific background and rationale for the investigation being reported	4-5
Objectives	3	State specific objectives, including any prespecified hypotheses	5
**Methods**			
Study design	4	Present key elements of study design early in the paper	5
Setting	5	Describe the setting, locations, and relevant dates, including periods of recruitment, exposure, follow-up, and data collection	5-7
Participants	6	(*a*) *Cohort study*—Give the eligibility criteria, and the sources and methods of selection of participants. Describe methods of follow-up<br>*Case-control study*—Give the eligibility criteria, and the sources and methods of case ascertainment and control selection. Give the rationale for the choice of cases and controls<br>*Cross-sectional study*—Give the eligibility criteria, and the sources and methods of selection of participants	6
		(*b*) *Cohort study*—For matched studies, give matching criteria and number of exposed and unexposed<br>*Case-control study*—For matched studies, give matching criteria and the number of controls per case	NA
Variables	7	Clearly define all outcomes, exposures, predictors, potential confounders, and effect modifiers. Give diagnostic criteria, if applicable	7, Figs1-2
Data sources/ measurement	8*	For each variable of interest, give sources of data and details of methods of assessment (measurement). Describe comparability of assessment methods if there is more than one group	6-7
Bias	9	Describe any efforts to address potential sources of bias	NA
Study size	10	Explain how the study size was arrived at	NA
Quantitative variables	11	Explain how quantitative variables were handled in the analyses. If applicable, describe which groupings were chosen and why	8
Statistical methods	12	(*a*) Describe all statistical methods, including those used to control for confounding	8
		(*b*) Describe any methods used to examine subgroups and interactions	NA
		(*c*) Explain how missing data were addressed	NA
		(*d*) *Cohort study*—If applicable, explain how loss to follow-up was addressed<br>*Case-control study*—If applicable, explain how matching of cases and controls was addressed<br>*Cross-sectional study*—If applicable, describe analytical methods taking account of sampling strategy	NA
		(*e*) Describe any sensitivity analyses	8

For peer review only - http://bmjopen.bmj.com/site/about/guidelines.xhtml

Continued on next page

**Results**			**Page**
Participants	13*	(a) Report numbers of individuals at each stage of study—eg numbers potentially eligible, examined for eligibility, confirmed eligible, included in the study, completing follow-up, and analysed	8
		(b) Give reasons for non-participation at each stage	NA
		(c) Consider use of a flow diagram	NA
Descriptive data	14*	(a) Give characteristics of study participants (eg demographic, clinical, social) and information on exposures and potential confounders	8-9, Fig3
		(b) Indicate number of participants with missing data for each variable of interest	NA
		(c) *Cohort study*—Summarise follow-up time (eg, average and total amount)	NA
Outcome data	15*	*Cohort study*—Report numbers of outcome events or summary measures over time	*9*
		Case-control study—Report numbers in each exposure category, or summary measures of exposure	*NA*
		Cross-sectional study—Report numbers of outcome events or summary measures	*9*
Main results	16	(*a*) Give unadjusted estimates and, if applicable, confounder-adjusted estimates and their precision (eg, 95% confidence interval). Make clear which confounders were adjusted for and why they were included	9-11
		(*b*) Report category boundaries when continuous variables were categorized	NA
		(*c*) If relevant, consider translating estimates of relative risk into absolute risk for a meaningful time period	NA
Other analyses	17	Report other analyses done—eg analyses of subgroups and interactions, and sensitivity analyses	10
**Discussion**			
Key results	18	Summarise key results with reference to study objectives	10-11
Limitations	19	Discuss limitations of the study, taking into account sources of potential bias or imprecision. Discuss both direction and magnitude of any potential bias	13-14
Interpretation	20	Give a cautious overall interpretation of results considering objectives, limitations, multiplicity of analyses, results from similar studies, and other relevant evidence	14-15
Generalisability	21	Discuss the generalisability (external validity) of the study results	14
**Other information**			
Funding	22	Give the source of funding and the role of the funders for the present study and, if applicable, for the original study on which the present article is based	2

*Give information separately for cases and controls in case-control studies and, if applicable, for exposed and unexposed groups in cohort and cross-sectional studies.

Note: An Explanation and Elaboration article discusses each checklist item and gives methodological background and published examples of transparent reporting. The STROBE checklist is best used in conjunction with this article (freely available on the Web sites of PLoS Medicine at http://www.plosmedicine.org/, Annals of Internal Medicine at http://www.annals.org/, and Epidemiology at http://www.epidem.com/). Information on the STROBE Initiative is available at www.strobe-statement.org.

For peer review only - http://bmjopen.bmj.com/site/about/guidelines.xhtml
